# A *de novo* transcriptome of the Malpighian tubules in non-blood-fed and blood-fed Asian tiger mosquitoes *Aedes albopictus*: insights into diuresis, detoxification, and blood meal processing

**DOI:** 10.7717/peerj.1784

**Published:** 2016-03-10

**Authors:** Carlos J. Esquivel, Bryan J. Cassone, Peter M. Piermarini

**Affiliations:** 1 Department of Entomology, The Ohio State University/Ohio Agricultural Research and Development Center, Wooster, OH, United States; 2 Department of Biology, Brandon University, Brandon, Manitoba, Canada

**Keywords:** Next generation sequencing, *De novo* transcriptome, Hematophagy, Malpighian tubule physiology, Diuresis, Xenobiotic detoxification, Purine metabolism

## Abstract

**Background.** In adult female mosquitoes, the renal (Malpighian) tubules play an important role in the post-prandial diuresis, which removes excess ions and water from the hemolymph of mosquitoes following a blood meal. After the post-prandial diuresis, the roles that Malpighian tubules play in the processing of blood meals are not well described.

**Methods.** We used a combination of next-generation sequencing (paired-end RNA sequencing) and physiological/biochemical assays in adult female Asian tiger mosquitoes (*Aedes albopictus*) to generate molecular and functional insights into the Malpighian tubules and how they may contribute to blood meal processing (3–24 h after blood ingestion).

**Results/Discussion.** Using RNA sequencing, we sequenced and assembled the first *de novo* transcriptome of Malpighian tubules from non-blood-fed (NBF) and blood-fed (BF) mosquitoes. We identified a total of 8,232 non-redundant transcripts. The Malpighian tubules of NBF mosquitoes were characterized by the expression of transcripts associated with active transepithelial fluid secretion/diuresis (e.g., ion transporters, water channels, *V*-type H^+^-ATPase subunits), xenobiotic detoxification (e.g., cytochrome P450 monoxygenases, glutathione *S*-transferases, ATP-binding cassette transporters), and purine metabolism (e.g., xanthine dehydrogenase). We also detected the expression of transcripts encoding sodium calcium exchangers, G protein coupled-receptors, and septate junctional proteins not previously described in mosquito Malpighian tubules. Within 24 h after a blood meal, transcripts associated with active transepithelial fluid secretion/diuresis exhibited a general downregulation, whereas those associated with xenobiotic detoxification and purine catabolism exhibited a general upregulation, suggesting a reinvestment of the Malpighian tubules’ molecular resources from diuresis to detoxification. Physiological and biochemical assays were conducted in mosquitoes and isolated Malpighian tubules, respectively, to confirm that the transcriptomic changes were associated with functional consequences. In particular, *in vivo* diuresis assays demonstrated that adult female mosquitoes have a reduced diuretic capacity within 24 h after a blood meal. Moreover, biochemical assays in isolated Malpighian tubules showed an increase in glutathione *S*-transferase activity and the accumulation of uric acid (an end product of purine catabolism) within 24 h after a blood meal. Our data provide new insights into the molecular physiology of Malpighian tubules in culicine mosquitoes and reveal potentially important molecular targets for the development of chemical and/or gene-silencing insecticides that would disrupt renal function in mosquitoes.

## Introduction

In mosquitoes, the renal (Malpighian) tubules produce urine via active transepithelial fluid secretion. This ‘primary’ urine consists mostly of Na^+^, K^+^, Cl^−^, and water, and provides a compartment for the excretion of metabolic and xenobiotic wastes. Additional modifications are made to the urine (e.g., water and/or solute reabsorption) in the hindgut before it is expelled from the animal. Thus, together the Malpighian tubules and hindgut of mosquitoes are the functional analogs of the mammalian kidney. In adult female mosquitoes, the Malpighian tubules play an especially important role in acute salt and water balance during and after engorgement with vertebrate blood. In particular, the tubules mediate the post-prandial diuresis, which rids mosquitoes of unwanted water and salts that are absorbed into their hemolymph after blood feeding. The diuresis begins while mosquitoes are still feeding on the host and continues for the next 1–2 h (Coast, 2009; Williams, Hagedorn & Beyenbach, 1983).

Over the past 30 years, numerous physiological and molecular studies on mosquito Malpighian tubules have uncovered a variety of mechanisms that regulate and mediate the post-prandial diuresis (e.g., ion transporters, neuroendocrine factors) (Beyenbach, 2003; Beyenbach, 2012; Beyenbach & Piermarini, 2011; Coast, 2009; Piermarini & Gillen, 2015). However, our understanding of how mosquito Malpighian tubules contribute to osmotic balance and blood meal processing after the post-prandial diuresis is limited. Presumably, the tubules contribute to the excretion of metabolic wastes, such as uric acid, that are produced and excreted by mosquitoes 12–72 h post-blood meal (Briegel, 1986; Von Dungern & Briegel, 2001).

To provide molecular insights into the putative functional contributions that Malpighian tubules make towards the metabolism of blood meals, our group recently performed a transcriptomic study on Malpighian tubules of the Asian tiger mosquito (*Aedes albopictus*) using single-end sequencing (i.e., RNA sequencing) (Esquivel, Cassone & Piermarini, 2014). In brief, we found general changes in transcript abundance that suggested the Malpighian tubules (1) decreased their capacity for diuresis, and (2) enhanced their capacity for detoxification and excretion of metabolic wastes (e.g., heme, ammonia) after a blood meal (Esquivel, Cassone & Piermarini, 2014). The Dow laboratory also recently performed a transcriptomic study on Malpighian tubules of blood-fed mosquitoes (Overend et al., 2015). Using microarrays, they found that the Malpighian tubules of the malaria mosquito *Anopheles gambiae* exhibited an upregulation of detoxification mechanisms at 3 h following a blood meal, but not a downregulation of diuretic mechanisms (Overend et al., 2015). Thus, the renal handling of blood meals may differ between culicine and anopheline mosquitoes, or the transcriptional changes to the diuretic mechanisms in *An. gambiae* may occur more slowly than in *Ae. albopictus*.

The goals of the present study were to improve upon our initial transcriptomic study by (1) establishing a *de novo* transcriptome for the Malpighian tubules of non-blood-fed and blood-fed *Ae. albopictus* using a paired-end RNA-Sequencing approach, (2) analyzing transcript expression in the Malpighian tubules of non-blood-fed mosquitoes, (3) confirming previous findings and generating new insights into changes in renal transcript expression that occur after a blood meal, and (4) validating whether transcriptomic changes in the Malpighian tubules after a blood meal manifest as functional changes in the mosquitoes and tubules. In brief, we have developed the first *de novo* transcriptome of Malpighian tubules from non-blood-fed and blood-fed mosquitoes, generated new insights into the molecular physiology of the Malpighian tubules in culcine mosquitoes, and confirmed that changes in renal transcript expression after blood feeding correlate with physiological and biochemical changes in mosquitoes and Malpighian tubules, respectively.

## Materials and Methods

### Mosquito colony rearing and maintenance

Eggs of *Ae. albopictus* (ALBOPICTUS, MRA-804, deposited by Sandra Allan) were obtained from the Malaria Research and Reference Reagent Resource Center (MR4) as part of BEI Resources Repository, NIAID, NIH. Eggs were hatched in tap water under vacuum at room temperature for two hours; the resulting larvae were raised in tap water at 28 °C with a 12 h:12 h light:dark cycle and fed pulverized TetraMin flakes (Melle, Germany). Adults were maintained under similar temperature and light conditions at 80% relative humidity and fed a 10% sucrose solution through cotton wicks. Only adult females (5–10 days post-eclosion) were used in the present study.

### Transcriptome assembly and analysis

#### Blood feeding and isolation of Malpighian tubules for cDNA library preparation

For a given experimental trial, 90 adult female mosquitoes were transferred from the main colony to two small containers (45 females per container) and starved for 24 h by removing their sucrose solution. After 24 h, one container of mosquitoes was provided with access to 10% sucrose for 30 min (non-blood-fed treatment, NBF) and the other was provided with access to heparinized rabbit blood (Hemostat Laboratories, Dixon, CA, USA) through a membrane feeder (Hemotek, Blackburn, UK) for 30 min. After 3 h, 12 h, or 24 h, mosquitoes were immobilized on ice for ∼15 min and the Malpighian tubules were isolated with fine forceps under Ringer solution (150 mM NaCl, 3.4 mM KCl, 1.7 mM CaCl_2_, 1.8 mM NaHCO_3_, 1.0 mM MgCl_2_, 5 mM glucose, and 25 mM HEPES; pH 7.1). The tubules were transferred immediately to ice-cold TRIzol Reagent (Life Technologies, Carlsbad, CA, USA) after isolation. Each replicate consisted of ∼200 Malpighian tubules isolated from 40 adult females. Twelve replicates were collected for NBF mosquitoes and 3 replicates each were collected for BF mosquitoes at 3 h, 12 h, and 24 h post-blood meal.

#### RNA isolation and cDNA synthesis

Immediately after a replicate of tubules was isolated, total RNA was extracted, cleaned, and purified as previously described (Esquivel, Cassone & Piermarini, 2014). The quality and concentration of the RNA were determined with an Experion Automated Electrophoresis System (Bio-Rad, Hercules, CA, USA) and a Qubit 2.0 Fluorometer (Life Technologies), respectively. The TruSeq DNA Sample Prep Kit V2, Set A and B (Illumina, San Diego, CA) was used for the synthesis of double-stranded cDNA libraries as previously described (Esquivel, Cassone & Piermarini, 2014). These libraries were non-strand specific, barcoded with unique indexed adapters, and consisted of ∼270 bp fragments. The quality and concentration of the double-stranded cDNA was determined with an Agilent 2100 Bioanalyzer High Sensitivity DNA Chip (Agilent Technologies, Santa Clara, CA, USA) and Qubit 2.0 Fluorometer (Life Technologies), respectively.

#### Sequencing of cDNA libraries

The 21 cDNA libraries were pooled to form a multiplexed library (18 nM each). This library was diluted to 36 fM and sequenced (paired-end reads of 100 bp in length) on a single lane of a flow cell using the Illumina HiSeq 2000 platform at The Ohio State University Comprehensive Cancer Center (Columbus, OH, USA). CASAVA (version 1.8.2) was used to demultiplex the sequencing data, and ‘basecall’ files were used to generate FASTQ files. The paired-end reads can be retrieved at the SRA (Sequence Read Archive) of the NCBI (National Center for Biotechnology Information) under the BioProject accession number PRJNA279095(experiment SRX964103).

The reads were trimmed, filtered, assembled, and annotated as described in the Supplemental Information 1, resulting in a total of 8,232 non-redundant contigs or transcripts (hereafter referred to as ‘*Ae. albopictus* transcripts’), which comprise the adult female *Ae. albopictus* Malpighian tubule transcriptome. This *de novo* transcriptome was deposited into the NCBI Transcriptome Shotgun Assembly (TSA) database under BioSample number SRS883933and accession number GCZT00000000.

#### Differential expression analysis

The transcripts from each of the 21 paired-end cDNA libraries were aligned onto the annotated *Ae. albopictus* Malpighian tubule transcriptome using the ‘Burrow-Wheeler Aligner’ version 1.2.3 (Li & Durbin, 2009). The SAM files generated were submitted to ‘SAM/BAM To Counts’ version 1.0.0 in the MCIC-Galaxy pipeline, for counting the reads aligned onto the *Ae. albopictus* transcripts. The ‘SAM/BAM To Counts’ output was submitted to the data package ‘DESeq2’ version 1.4.5 (Love, Huber & Anders, 2014), imported from Bioconductor (Gentleman et al., 2004) and customized onto the R programming language. The number of reads from the NBF libraries were pooled together and used as a universal control for comparison with each of the BF libraries. The relative expression changes of transcripts between the NBF control and a BF library were considered statistically significant if the FDR-adjusted *P*-value was less than 0.05.

#### Functional clustering analysis of transcripts

Transcripts from the NBF libraries with reads per kilobase per million mapped reads (RPKM) values >0 were subjected to a Database for Annotation, Visualization and Integrated Discovery (DAVID, version 6.7) functional clustering analysis (Huang da, Sherman & Lempicki, 2009) as described in the Supplemental Information 1. Transcripts that were differentially expressed between the BF and NBF libraries were also subjected to a DAVID analysis. Only the functional clusters with enrichment values >1.3 (equivalent to a *P*-value < 0.05) were retained (Esquivel, Cassone & Piermarini, 2014).

### Functional assays

#### In vivo diuresis assay

The diuretic capacities of NBF and BF adult female *Ae. albopictus* mosquitoes were determined using a previously described assay (Raphemot et al., 2013). In brief, NBF and BF mosquitoes were prepared as described in “*Blood feeding and isolation of Malpighian tubules for cDNA library preparation*.” At 3 h, 12 h, and 24 h after feeding, five mosquitoes were immobilized on ice and their hemolymph was injected with 900 nl (100 nl/sec) of a phosphate-buffered saline (PBS) using a Nanoject II nanoliter injector (Drummond Scientific, Broomall, PA, USA). The PBS consisted of 137 mM NaCl, 2.7 mM KCl, 10 mM Na_2_HPO_4_, and 2 mM KH_2_PO_4_ (pH 7.5) (Fisher Scientific, Hampton, NH, USA). Immediately after injection, the 5 mosquitoes were transferred to a graduated packed-cell volume (PCV) tube (MidSci, St. Louis, MO, USA) and placed into an incubator at 28 °C. After 30 min, the mosquitoes were removed and the PCV tube was centrifuged to collect the urine in the graduated column at the bottom of the tube. The amount of urine excreted was measured visually from the gradations. At least 9 replicates of 5 mosquitoes each were performed for each treatment. The baseline excretion volumes of uninjected NBF and BF mosquitoes for each time point were also determined and subtracted from those of the corresponding injected mosquitoes.

#### Glutathione S-transferase (GST) activity

GST activity in Malpighian tubules was measured by adopting elements of standard biochemical assays (Brogdon & Barber, 1990; Habig, Pabst & Jakoby, 1974; Hemingway, 1998). This assay is based on the GST-mediated conjugation of reduced glutathione (GSH) to 1-chloro-2,4-dinitrobenzene (CDNB) to generate 3-(2-chloro-4-nitrophenil)-glutathione; CDNB absorbs light at 340 nm. The amount of CDNB (nmol) conjugated is calculated based on (1) the rate of the absorbance increase at 340 nm, and (2) the extinction coefficient of CDNB (0.00503 µM^−1^) (Habig, Pabst & Jakoby, 1974).

In the present study, NBF and BF mosquitoes were prepared and ∼100 Malpighian tubules were isolated from 20 females, as described in “*Blood feeding and isolation of Malpighian tubules for cDNA library preparation*.” The Malpighian tubules were stored at −80 °C until the day of the assay. At least nine biological replicates were collected for each treatment/time point. Immediately before the assay, the Malpighian tubules were thawed on ice and homogenized with a plastic pestle in 100 µl of a potassium phosphate buffer, which consisted of 100 mM potassium phosphate (pH 7.0), and 0.1% Triton X-100 (Fisher Scientific, Hampton, NH, USA). The crude tubule lysates were centrifuged at 10,000 g for 15 min at 4 °C. The supernatant was transferred to a clean 1.5 ml microcentrifuge tube on ice.

For the analysis of a single sample, the following were combined in the well of a UV transparent 96-well microtiter plate (Thermo Fisher Scientific, Waltham, MA, USA): 4 µl of the tubule supernatant, 186 µl of 200 mM GSH (Sigma-Aldrich, St. Louis, MO, USA), and 10 µl of 20 mM CDNB (Acros Organics, Geel, Belgium). GSH and CDNB were diluted in 17 MΩ-water and 100% ethanol, respectively. The GSH was dissolved in PBS. On a given microplate, each sample was represented in triplicate. Immediately after adding the CDNB, the microtiter plate was placed into a Multiskan GO Microplate Spectrophotometer (Thermo Scientific, Waltham, MA, USA) and submitted to a ‘slow’ mixing protocol for 10 s at 25 °C. Thereafter, the absorbance at 340 nm was measured every minute for six minutes. The calculated GST activity (nmol CDNB/min) of each sample was normalized to its total protein content, which was measured with a Bradford protein assay (Bio-Rad, Hercules, CA, USA).

#### Uric acid assay

The Eton^®^ Uric Assay Kit (Eton Bioscience, Inc., San Diego, CA, USA) was used to measure the concentration of uric acid in isolated Malpighian tubules of NBF and BF mosquitoes. Malpighian tubules were isolated from NBF and BF mosquitoes as described above. Each replicate consisted of ∼100 isolated Malpighian tubules from 20 females. In brief, isolated tubules were homogenized in 300 µl of dH_2_O and 50 µl of the homogenate was analyzed for uric acid in UV transparent 96-well plates (Thermo Fisher Scientific) following the manufacturer’s protocol. On a given microplate, each sample was represented in duplicate. For each sample, the uric acid content (mmol uric acid) was normalized to its total protein content, which was measured with a Bradford protein assay (Bio-Rad).

#### Statistical analyses for functional assays

For the diuresis, GST, and uric acid assays, the data from the NBF and BF treatments were analyzed via a one-way ANOVA with a Newman-Keuls posttest using GraphPad Prism 6.0 (San Diego, CA).

## Results and Discussion

### Sequencing, assembly, annotation, and mapping of *de novo* transcriptome

A total of 21 Malpighian tubule cDNA libraries (12 from NBF mosquitoes, 9 from BF mosquitoes) were sequenced in the present study, resulting in ∼435 million raw reads. After data preprocessing, ∼19% of the reads were omitted, resulting in ∼351 million reads. The free-reference assembly was performed on ∼115 million of these trimmed reads derived from 7 representative cDNA libraries (see Supplemental Information 1 for details), resulting in 28,189 non-redundant contigs. Using this initial assembly of filtered contigs as a reference, all of the trimmed reads from the 21 cDNA libraries were mapped and subjected to a BLASTn analysis for further refinement (see Supplemental Information 1 for details), resulting in 8,232 non-redundant ‘*Ae. albopictus* transcripts.’

The *Ae. albopictus* transcripts had the greatest number of unique matches with the *Ae. aegypti* transcriptome (*n* = 917) compared to the *C. quinquefasciatus* (*n* = 95), *An. gambiae* (*n* = 50), and *D. melanogaster* (*n* = 10) transcriptomes (Fig. S1), which is consistent with the relative degrees of phylogenetic separation among these dipteran species (Chen et al., 2015; Reidenbach et al., 2009; Wiegmann et al., 2011). The distribution by length of the *Ae. albopictus* transcripts showed that ∼44% of the contigs had a length between 300 bp and 1,300 bp (Fig. S2), which indicates that the contig refinement did not cause a bias of the contig distribution (O’Neil & Emrich, 2013). This pattern is comparable with that for other mosquito *de novo* transcriptomes (Crawford et al., 2010; Huang, Poelchau & Armbruster, 2015; Padrón et al., 2014; Poelchau et al., 2011; Zhu et al., 2014).

The majority of the transcript annotations (67%) were made with high confidence as indicated by their exceptional *E*-value (<10^−180^). Furthermore, more than 32% of the annotated transcripts had an ortholog hit ratio (OHR) between 0.9 and 1.1 (mode of 1.0) (Fig. S3), which is comparable to the OHR reported in other insect transcriptomes (Ewen-Campen et al., 2011; O’Neil & Emrich, 2013; Van Belleghem et al., 2012) and suggests that the transcriptome was correctly assembled (O’Neil & Emrich, 2013; O’Neil et al., 2010). All together, the above data suggest that we have generated a robust, high-quality *de novo* transcriptome—the first derived from the Malpighian tubules of NBF and BF mosquitoes.

### Comparisons of Malpighian tubule cDNA libraries from NBF and BF mosquitoes

**Figure 1:**
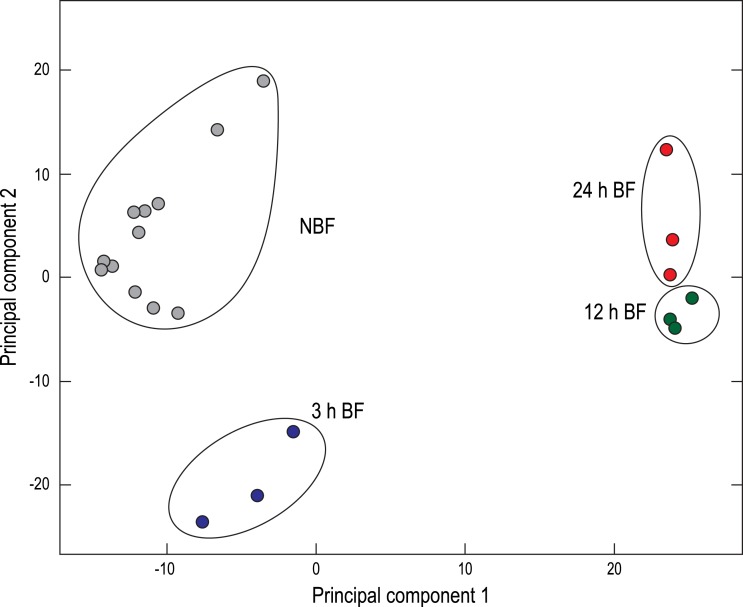
Principal component analysis (PCA) of the paired-end Malpighian tubule cDNA libraries. Each circle represents an individual cDNA library from the indicated treatment. NBF, non-blood-fed; BF, blood-fed.

A two-dimensional principal component analysis (PCA) was generated to visualize the relationships among the Malpighian tubule cDNA libraries derived from NBF and BF mosquitoes (Fig. 1). The NBF libraries clustered in the upper/middle left, the 3 h BF libraries clustered in the lower left, and the 12 h and 24 h BF libraries clustered near one another in the upper/middle right (Fig. 1). This spatial distribution is similar to what we have observed in a previous study (Esquivel, Cassone & Piermarini, 2014) and confirms that a blood meal influences the global expression of transcripts in Malpighian tubules in a time-dependent manner.

**Table 1:** Numbers of *Ae. albopictus* transcripts differentially expressed in Malpighian tubules of 3 h, 12 h, and 24 h BF mosquitoes, relative to those of NBF controls.

Time point	Upregulated	Downregulated	Total
3 h	1,862	1,910	3,772
12 h	1,673	1,677	3,350
24 h	1,489	1,724	3,213

DESeq analysis of the cDNA libraries revealed that a blood meal elicited the differential expression of over 3,200 transcripts in Malpighian tubules at each time point (relative to NBF controls) with approximately equal numbers of upregulated and downregulated transcripts (Table 1). This degree of differential expression (1) represents a substantial improvement from our previous study in which we detected less than 1,235 differentially-expressed transcripts at each time point (Esquivel, Cassone & Piermarini, 2014) and (2) is consistent with results of a recent transcriptomic study in adult female *Ae. albopictus* that found ∼2,000 differentially-expressed transcripts at 26–28 h after a blood meal (Huang, Poelchau & Armbruster, 2015). A recent microarray study in the Malpighian tubules of *An. gambiae* found that >1,200 transcripts were differentially expressed at 3 h after blood feeding, with the vast majority being upregulated (Overend et al., 2015). Thus, both species show similar degrees of transcript enrichment in Malpighian tubules at 3 h after a blood meal, but *Ae. albopictus* is novel from *An. gambiae* in exhibiting a reciprocal degree of transcript depletion at this time point (Table 1).

Figure S4 shows that most of the novel changes in transcript expression occur at 3 h after a blood meal, followed by attenuations at 12 h and 24 h. In total, for all three time periods, 3,973 non-redundant transcripts were differentially expressed after blood feeding; this number represents a substantial improvement from the 1,857 non-redundant transcripts detected in our previous study (Esquivel, Cassone & Piermarini, 2014). Thus, by developing a *de novo* transcriptome of the Malpighian tubules, we have revealed that a blood meal has an even larger quantitative impact on renal transcript expression than previously recognized.

### Functional pathway analysis of transcripts expressed in Malpighian tubules of NBF mosquitoes

To obtain insights into functional pathways that were enriched among the transcripts expressed in the Malpighian tubules of NBF mosquitoes, we conducted a DAVID functional clustering analysis. A total of 47 functional clusters were significantly enriched, which we manually categorized into five general themes based on the predicted functions of the proteins encoded by the transcripts within the clusters: (1) Transcription and translation (17 clusters), (2) Protein sorting and trafficking (7 clusters), (3) Proteolysis (4 clusters), (4) ATP metabolism (4 clusters), and (5) Redox and detoxification (3 clusters) (Table 2). Twelve of the 47 clusters were not categorized (‘Uncategorized,’ Table S1).

**Table 2:** Categorization of the DAVID functional clusters enriched among the transcripts expressed in the Malpighian tubules of NBF mosquitoes. See Table S2 for the specific transcripts in these clusters. Category names are bolded and italicized; the functional clusters are listed immediately below the category name in regular text.

	EV^*^
***Transcription and translation***
Ribosome	4.32
Translation initiation factor	4.21
Regulation of mRNA processing	3.78
Basal transcription factors	2.29
Translation termination	2.07
Bromodomain	1.76
Transcription factor complex	1.74
RNA polymerase	1.66
tRNA processing	1.46
Aminoacyl-tRNA biosynthesis	1.45
Chaperone/heat shock protein DnaJ	1.44
Negative regulation of gene expression	1.42
Histone acetyltransferase activity	1.38
Translation initiation factor activity	1.36
Chromatin modification	1.36
mRNA catabolic process	1.31
Negative regulation of protein process	1.3
***Protein sorting and trafficking***	
Nuclear transport	24.04
Protein transport 1	3.49
Protein transport 2	3.36
Vesicle-mediated transport 1	2.88
Vesicle-mediated transport 2	2.45
Endoplasmic reticulum	2.31
Calcium/phospholipid-binding	1.38
***Proteolysis***	
Ubiquitin-dependent protein catabolic	3.2
Ubiquitin mediated proteolysis	2.88
Ubl conjugation pathway	1.87
Proteasome	1.58
***ATP metabolism***	
ATP binding	3.46
ATPase activity	2.92
Mitochondrial membrane	1.88
Mitochondrial substrate carrier	1.58
***Redox and detoxification***	
Thioredoxin	24.88
Ommochrome biosynthetic process	1.9
Aldehyde dehydrogenase	1.34

**Notes.**

* Enrichment value.

An inspection of the transcripts listed in the functional clusters of the ‘Transcription and translation’, ‘Protein sorting and trafficking,’ and ‘Proteolysis’ categories (Table S2) suggests that the Malpighian tubules of NBF mosquitoes are enriched with the molecular machinery for the: (1) synthesis, splicing, and maturation of mRNAs; (2) synthesis, post-translational modification, and sorting/trafficking of new proteins; and (3) degradation and recycling of old/damaged proteins. Furthermore, the transcripts listed in the functional clusters of the ‘ATP metabolism’ and ‘Redox and detoxification’ category (Table S2) suggest that the Malpighian tubules of NBF mosquitoes are enriched with the molecular machinery for (1) the synthesis of ATP and (2) limiting cellular damage and oxidative stress from reactive oxygen species.

### Functional pathway analysis of transcripts differentially expressed in Malpighian tubules after blood feeding

To gain insights into putative broad, functional transitions occurring in the Malpighian tubules after blood feeding, we conducted a DAVID functional cluster analysis on the differentially-expressed transcripts at each time point (3 h, 12 h, 24 h). This analysis revealed a total of 71 functional clusters that were significantly-enriched among the upregulated transcripts and 57 functional clusters that were significantly-enriched among the downregulated transcripts. We manually categorized 116 of these clusters according to their time point, direction of regulation (up or down), and general functional theme (Table 3).

**Table 3:** The number of up- and downregulated functional clusters within each general functional category for each time point after a blood meal. Category names are bolded and italicized. The dominant direction of regulation (up or down) is bolded. See Table S3 for the functional clusters within each category, and Tables S4–S7 for the transcripts associated with each functional cluster.

	3 h BF		12 h BF		24 h BF	
	*Up*	*Down*	*Up*	*Down*	*Up*	*Down*
***Transcription and Translation***	**10**	0	0	**5**	0	**13**
***Protein Sorting and Trafficking***	**7**	0	**7**	2	**9**	1
***Proteolysis***	**5**	0	**2**	0	**2**	0
***ATP metabolism***	2	**13**	0	**6**	2	**11**
***Redox and Detoxification***	**5**	0	**5**	0	**9**	0

In four of the five general categories, we found consistent directional trends (Table 3). That is, from 3 h to 24 h, there was a dominant (1) downregulation of molecular pathways associated with ATP metabolism, and (2) upregulation of molecular pathways associated with ‘Protein sorting and trafficking,’ ‘Proteolysis,’ and ‘Redox and detoxification.’ Thus, in the first 24 h after a blood meal, the tubules appear to decrease their molecular capacity for ATP synthesis and active transport while increasing their capacity for protein turnover, antioxidant production, and xenobiotic detoxification, which verifies findings from our previous study (Esquivel, Cassone & Piermarini, 2014).

In contrast, a dynamic trend was found in the molecular pathways associated with ‘Transcription and translation’ (Table 3). That is, at 3 h after a blood meal, these pathways were upregulated, whereas at 12 h and 24 h they were downregulated. Thus, the molecular capacity for synthesizing mRNA and proteins is initially enhanced, but is then suppressed at the latter time points. This finding adds to the results of our previous study, which had only documented a downregulation in the molecular capacity for mRNA translation at 12 h and 24 h after a blood meal (Esquivel, Cassone & Piermarini, 2014).

In the remaining sections, we focus on the expression of transcripts related to active transepithelial fluid secretion/diuresis, xenobiotic detoxification and excretion, and purine metabolism. In particular, we describe the expression of these transcripts in Malpighian tubules of NBF mosquitoes and how they change after a blood meal. In addition, we employ functional assays to determine whether the changes in transcript expression noted after a blood meal are correlated with biochemical and physiological changes in Malpighian tubules and mosquitoes, respectively.

### Mechanisms of active transepithelial fluid secretion/diuresis

Figure S5 summarizes the mechanisms of transepithelial fluid secretion in mosquito Malpighian tubules. In brief, fluid secretion is ultimately driven by the activity of a *V*-type H^+^-ATPase in the apical brush border of principal cells (Beyenbach, 2001; Beyenbach, Pannabecker & Nagel, 2000; Weng et al., 2003), which establishes the electrochemical gradients necessary for transporting Na^+^, K^+^, Cl^−^, and water across the Malpighian tubule epithelium through a variety of ion transporters/channels and water channels (aquaporins, AQPs) in principal and stellate cells (Fig. S5). In the cases of Cl^−^ and water, transepithelial transport may also occur between cells via a paracellular pathway formed by septate junctions (‘SJ’ in Fig. S5). The activity of the *V*-type H^+^-ATPase is dependent upon the availability of intracellular ATP, which is synthesized by mitochondria in the apical microvilli (Figs. S5 and S6).

In the Malpighian tubules of NBF *Ae. albopictus*, we detected the expression of numerous transcripts that encode ion and water transport mechanisms (e.g., *V*-type H^+^-ATPase subunits, AQPs, cation proton antiporters, cation chloride cotransporters, inward rectifier K^+^ channels) and key elements of mitochondrial ATP synthesis (e.g., electron transport chain complexes, ATP synthase subunits, ADP/ATP transporters) (Table S8). In brief, the overall qualitative and quantitative patterns of the ion and water transport mechanisms were very similar to those described for the Malpighian tubules of adult female *An. gambiae* and adult *D. melanogaster*, consistent with the notion that there is a highly-conserved signature of transcript expression common to ion-transporting epithelia among dipteran species (Chintapalli et al., 2013; Overend et al., 2015). Below, we focus on novel insights generated by the present study.

#### Na^+^∕Ca^2+^ exchangers

To our surprise, the most abundant transcript encoding an ion transporter in the Malpighian tubules of NBF *Ae. albopictus* was that for a putative K-dependent Na^+^∕Ca^2+^ exchanger (NCKX) of the SLC24 superfamily (Table 4). Notably, NCKX1 was the fifth highest-expressed transcript in the tubules of NBF mosquitoes and over 3 times more abundant than the most-highly expressed subunit of the *V*-type H^+^-ATPase (i.e., ‘Locus135v1rpkm1149.21,’ subunit G, in Table S8). In addition, the transcripts for another NCKX (NCKX2) and two putative Na^+^∕Ca^2+^ exchangers (NCXs) of the SLC8 superfamily were expressed in tubules of NBF *Ae. albopictus*, but were of much lower abundance compared to NCKX1 (Table 4). In the Malpighian tubules of adult female *An. gambiae*, the NCKX1 ortholog (AGAP010975) is highly expressed and enriched (http://Moztubules.org; http://mozatlas.gen.cam.ac.uk).

**Table 4:** List of transcripts encoding putative Na^+^∕Ca^2+^ exchangers expressed in the Malpighian tubules of NBF mosquitoes.

*Ae. albopictus transcript ID*	Sequence description	*Ae. aegypti* ortholog	Mean RPKM value
Locus79v2rpkm1526.34	NCKX1	AAEL004805-RA	2,814
Locus954v1rpkm144.28	NCKX2	AAEL004814-RA	46
Locus11776v1rpkm7.43	NCX1	AAEL013788-RA	3
Locus14004v1rpkm5.50	NCX2	AAEL012480-RA	2

To date, a NCKX or NCX is not included in the current model of transepithelial fluid secretion in Malpighian tubules of mosquitoes (Fig. S5). In vertebrates, NCKXs and NCXs play critical roles in regulating concentrations of intracellular Ca^2+^ by exporting Ca^2+^ from the cytosol (Khananshvili, 2013; Schnetkamp, 2013). In mosquito Malpighian tubules, kinin diuretic peptides utilize intracellular Ca^2+^ as a second messenger (Pietrantonio et al., 2005; Yu & Beyenbach, 2002). Thus NCKXs and NCXs could potentially play an important role in (1) maintaining low intracellular Ca^2+^ prior to kinin activation and/or (2) returning intracellular Ca^2+^ to normal levels after kinin stimulation. It is also possible that these transporters contribute to the electrogenic entry of Na^+^ into the epithelium, assuming that they localize to the basolateral membrane. Elucidating the localization and functional role(s) of NCKXs and NCXs in mosquito Malpighian tubules should be a priority of future studies.

#### GPCRs

At least three neuroendocrine peptides regulate the rates of transepithelial fluid secretion in mosquito Malpighian tubules: diuretic hormone 31 (DH_31_), DH_44_, nin (Beyenbach & Piermarini, 2009; Coast, 2007; Coast, 2009). DH_31_ is also known as the mosquito natriuretic factor/peptide, which was first identified and characterized in the Malpighian tubules of *Ae. aegypti* by the Beyenbach laboratory (Beyenbach & Petzel, 1987; Petzel, Hagedorn & Beyenbach, 1985; Petzel, Hagedorn & Beyenbach, 1986; Sawyer & Beyenbach, 1985).

In the Malpighian tubules of NBF *Ae. albopictus*, we detected the expression of transcripts encoding putative DH_31_ and kinin GPCRs, but not one encoding a putative DH_44_ receptor (Table 5). In Malpighian tubules of *Ae. aegypti* and *An. gambiae*, transcripts for DH_44_ receptors are expressed (Jagge & Pietrantonio, 2008; Overend et al., 2015), which suggests that the endocrine regulation of fluid secretion in *Ae. albopictus* may differ from other mosquitoes. The most abundant GPCR in the tubules of NBF *Ae. albopictus* was a putative tachykinin receptor (Table 5). To our knowledge, the effects of tachykinin on Malpighian tubule function in mosquitoes are unknown, but in Malpighian tubules of locusts (*Locusta migratoria*, *Schistocerca gregaria*) and a lepidopteran (*Manduca sexta*), tachykinin promotes diuresis (Johard et al., 2003; Skaer et al., 2002).

**Table 5:** List of transcripts encoding putative G protein-coupled receptors (GPCRs) in the Malpighian tubules of NBF mosquitoes.

*Ae. albopictus* transcript ID	Sequence description	*Ae. aegypti* ortholog	Mean RPKM value
Locus247v7rpkm49.48_PRE	Neurokinin/Tachykinin Family	AAEL017341-RA	30
Locus1606v1rpkm78.86	Orphan/Putative Class D Family	AAEL001782-RA	23
Locus1029v1rpkm131.51	DH_31_ receptor	AAEL010043-RA	23
Locus2721v1rpkm43.27	Kinin receptor	AAEL006636-RA	14
Locus2557v1rpkm46.90	Predicted GPCR	AAEL010852-RA	14
Locus4349v1rpkm23.59	Galanin/Allatostatin Family	AAEL012920-RA	14
Locus4401v6rpkm9.12	HE6-like Family	AAEL017167-RA	12
Locus3331v1rpkm32.68	Methuselah Family	AAEL011521-RA	11
Locus7231v1rpkm13.12	Putative GPCR	AAEL013430-RA	9
Locus3268v1rpkm33.74	Melatonin Family	AAEL001606-RA	8
Locus11929v1rpkm7.29	Methuselah Family	AAEL000811-RA	7
Locus4472v1rpkm22.91	Methuselah Family	AAEL010980-RA	7
Locus106v39rpkm9.78_PRE	Predicted GPCR	AAEL002694-RA	6
Locus13075v1rpkm6.26	Growth Hormone Releasing Hormone Family	AAEL017335-RA	5
Locus8260v1rpkm11.30	Frizzled/Smoothened Family	AAEL006832-RA	4
Locus6828v1rpkm13.93	Frizzled/Smoothened Family	AAEL006669-RA	4
Locus9392v1rpkm9.89	Orphan/Putative Class B Family	AAEL000602-RA	4
Locus7819v1rpkm12.04	GPCR Methuselah Family	AAEL011526-RA	3
Locus8376v1rpkm11.16	Orphan/Putative Class B Family	AAEL001724-RA	3
Locus11502v1rpkm7.70	Methuselah Family	AAEL010982-RA	3
Locus10726v1rpkm8.41	Putative GPCR	AGAP008237-RA	2

We detected the expression of at least 16 other transcripts encoding putative GPCRs in the Malpighian tubules of NBF *Ae. albopictus* (Table 5). Some encode receptors for unknown ligands (i.e., orphaned receptors) or ligands not previously studied in mosquito Malpighian tubules, such as allatostatin. Notably, five transcripts encode putative Methusaleh GPCRs (Table 5); these receptors are linked to reproduction, aging, and stress resistance in *D. melanogaster* (Araújo et al., 2013; Caers et al., 2012). Moreover, two transcripts encode putative frizzled receptors (Table 5), which play key roles in Wnt signaling and tissue development/morphogenesis in *D. melanogaster* (Hanlon & Andrew, 2015). Thus, GPCRs are likely involved in regulating a wide range of physiological processes in mosquito Malpighian tubules, beyond their conventional roles in salt and water balance.

#### Septate junctional proteins

Septate junctions are hypothesized to play roles in the paracellular transport of Cl^−^ and H_2_O in mosquito Malpighian tubules (Fig. S5) and likely play important epithelial barrier functions. In insects, there are two types of septate junctions. Pleated septate junctions (pSJs) are typically found in tissues of ectodermal origin (e.g., epidermis, hindgut), whereas smooth septate junctions (sSJs) are typically found in tissues of endodermal origin (e.g., midgut). Malpighian tubules are exceptional in that they are derived from ectoderm, but contain sSJs (Izumi & Furuse, 2014).

In *D. melanogaster*, several transmembrane proteins have been shown to play key roles in the formation of pSJs, including claudin-like proteins (e.g., megatrachea, sinuous, and kune-kune), fasciclins (i.e., fas2 and fas3), gliotactin, lachesin, macroglobulin complement-related protein, melanotransferrin, neurexin IV, neuroglian, and subunits of the *α* and *β* subunits of the Na,K-ATPase (Izumi & Furuse, 2014). One fasciclin (fas3) is also a component of sSJs in the midgut of *D. melanogaster* (Izumi, Yanagihashi & Furuse, 2012). The only transmembrane proteins known to be specifically-associated with sSJs in *D. melanogaster* are snakeskin and mesh, which are expressed in the midgut and Malpighian tubules (Izumi & Furuse, 2014; Yanagihashi et al., 2012).

**Table 6:** List of transcripts encoding putative septate junctional transmembrane proteins in the Malpighian tubules of NBF mosquitoes.

*Ae. albopictus* transcript ID	Sequence description	*Ae. aegypti* ortholog	Mean RPKM value
Locus250v1rpkm562.31	snakeskin	AAEL002578-RA	107
Locus1140v1rpkm116.60	fasciclin	AAEL000540-RA	50
Locus1140v1rpkm116.60	fas3-like	AAEL000540-RA	50
Locus1755v4rpkm22.04	mesh	AAEL005432-RB	34
Locus1140v2rpkm24.99	fasciclin	AAEL000540-RB	19
Locus1140v2rpkm24.99	fas3-like	AAEL000540-RB	19
Locus1838v1rpkm68.02	fasciclin	AAEL000541-RB	17
Locus1838v1rpkm68.02	fas3-like	AAEL000541-RB	17
Locus1755v3rpkm22.27	mesh	AAEL005432-RA	12
Locus4693v1rpkm21.61	midline fasciclin	AAEL008657-RA	12
Locus3975v1rpkm26.55	fasciclin ii (fas ii)	AAEL009173-RA	10
Locus5066v1rpkm19.75	neuroglian, cell adhesion molecule	AAEL008340-RA	5
Locus5535v1rpkm17.83	gliotactin	AAEL009055-RA	5
Locus5141v1rpkm19.35	sinuous, conserved hypothetical protein	AAEL005218-RC	5
Locus10765v1rpkm8.39	megatrachea/pickel, conserved hypothetical protein	AAEL005228-RA	3
Locus6613v1rpkm14.50	neurexin iv	AAEL005321-RA	3
Locus12312v1rpkm6.92	melanotransferrin	AAEL011949-RA	3
Locus6507v1rpkm14.79	lachesin	AAEL009295-RA	2
Locus13560v1rpkm5.83	macroglobulin	AAEL012267-RA	1

As shown in Table 6, snakeskin and mesh were among the most abundantly-expressed transcripts encoding putative transmembrane sSJ proteins in Malpighian tubules of NBF *Ae. albopictus*. Moreover, several transcripts encoding fasciclins were abundantly expressed, suggesting a potential role of these proteins in the formation of sSJs in mosquito Malpighian tubules. Transcripts encoding claudin-like proteins and other key elements of pSJs (e.g., gliotactin, neurexin IV) were of lower abundance. Although transcripts encoding *α* and *β* subunits of the Na,K-ATPase were also abundant (Table S8), evidence for a structural role of these subunits in septate junctions of Malpighian tubules is lacking. The *α* subunit localizes to the basolateral membrane of stellate cells and ouabain inhibits transepithelial fluid secretion and ATPase activity in isolated Malpighian tubules (Hine et al., 2014; Patrick et al., 2006; Tiburcy, Beyenbach & Wieczorek, 2013), which suggests that the Na,K-ATPase primarily participates in membrane transport.

### Changes after blood feeding

After mosquitoes ingested a blood meal, the expression of transcripts associated with active transepithelial fluid secretion exhibited a prevailing trend of downregulation in Malpighian tubules. Notably, at each time point, at least 14 transcripts encoding subunits of the *V*-type H^+^-ATPase were downregulated and only one or two were upregulated (Table S9). Moreover, transcripts encoding several key ion transporters/channels, such as cation proton antiporters, inward rectifier K^+^ (Kir) channel subunits, and AQPs, were significantly downregulated, along with those encoding gap junctional proteins (innexins) carbonic anhydrases, and the aforementioned NCKX and NCX transcripts. Among these mechanisms, the majority of the differentially-expressed transcripts were downregulated at 3 h (5 up, 22 down), 12 h (10 up, 25 down), and 24 h (10 up, 22 down) (Table S9). At 3 h and 24 h, similar trends were also apparent in the transcripts encoding subunits of electron transport chain complexes, *F*-type ATP synthase subunits, and an ADP/ATP carrier protein (Table S9). Taken together, these data suggest that within 24 h after a blood meal, the tubules decrease their molecular capacity for active transepithelial fluid secretion and diuresis, which is consistent with the results of our previous study (Esquivel, Cassone & Piermarini, 2014).

**Figure 2:**
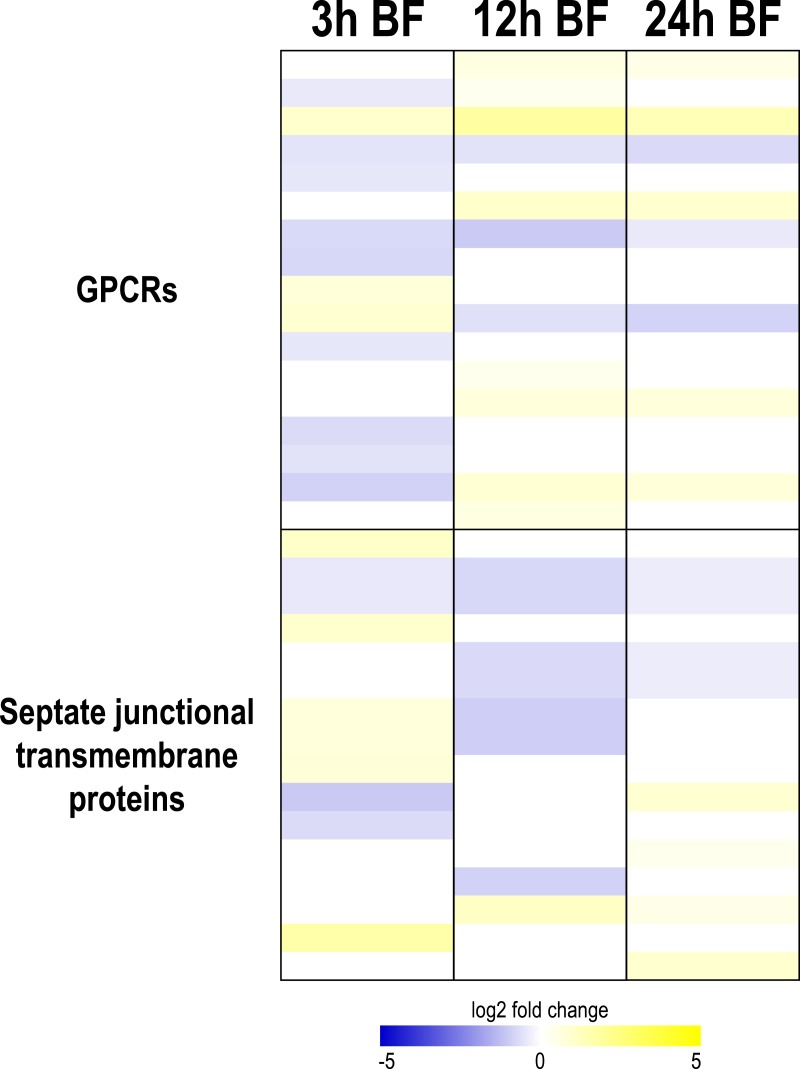
Heat map showing differential expression of transcripts encoding G protein-coupled receptors (GPCRs) and transmembrane proteins associated with septate junctions. Specific transcripts are listed in Table S9.

Transcripts encoding GPCRs showed a variable pattern of differential expression following blood feeding (Fig. 2, Table S9). Most transcripts were downregulated at 3 h after a blood meal (3 up, 9 down), whereas most were upregulated at 12 h (8 up, 3 down) and 24 h (5 up, 3 down) (Fig. 2, Table S9). Notably, the DH_31_ and kinin receptors showed opposite patterns of regulation after a blood meal (Table S9). That is, the kinin receptor, which enhances the secretion of NaCl, KCl, and water, showed a concerted downregulation, whereas the DH_31_ receptor, which enhances the secretion of NaCl and water, showed a concerted upregulation. It is unclear why transcripts for these receptors would be regulated in opposite directions, but the results may suggest a previously unconsidered role of the DH_31_ receptor in regulating the renal processing of blood meals after the post-prandial diuresis has concluded.

Transcripts encoding putative transmembrane septate-junctional proteins also exhibited a variable pattern of differential expression after a blood meal (Fig. 2, Table S9). Most transcripts were upregulated at 3 h after a blood meal (6 up, 4 down), whereas most were downregulated at 12 h (1 up, 7 down). At 24 h, similar numbers of transcripts were up- and downregulated (4 up, 4 down) (Fig. 2, Table S9). These results suggest that the septate junctions in Malpighian tubules may undergo a molecular remodeling during blood meal processing, which may affect the permeability of the paracellular pathway to H_2_O, Cl^−^, or other solutes (e.g., organic anions and cations).

### Diuresis assays

**Figure 3:**
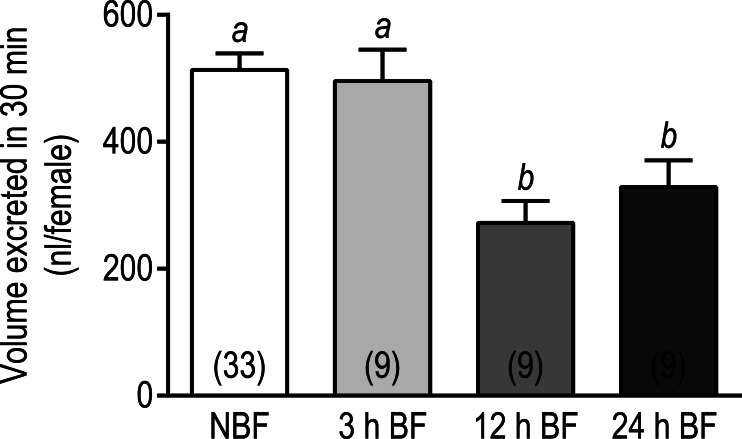
Effects of a blood meal on the diuretic capacity of adult female *Ae. albopictus*. Values are means ± SEM, based on the number of independent trials in parentheses. Lowercase letters indicate statistical difference as determined by a one-way ANOVA with a Newman-Keuls posttest (*P* < 0.05).

To determine whether the overall downregulation of transcripts associated with transepithelial fluid secretion in Malpighian tubules was associated with an effect on the diuretic capacity of mosquitoes, we conducted *in vivo* diuresis assays in adult female mosquitoes at 3 h, 12 h, and 24 h after a blood meal; NBF mosquitoes were used as controls. In brief, the hemolymph of mosquitoes was injected with 900 nl of a phosphate-buffered saline and the amount of urine excreted within 30 min was measured. As shown in Fig. 3, the volume of urine excreted by the 3 h BF mosquitoes (495.6 ± 49.5 nl) was not significantly different from that of NBF mosquitoes (513.3 ± 25.8 nl). However, the 12 h and 24 h BF mosquitoes both excreted significantly lower volumes of urine compared to the NBF and 3 h BF mosquitoes (12 h BF = 272.2 ± 34.6 nl; 24 h BF = 328.7 ± 42.1 nl) (Fig. 3). Although these data are consistent with the downregulation of transcripts associated with transepithelial fluid secretion in Malpighian tubules 3–24 h after a blood meal, we cannot rule out that other factors contributed to the observed results, such as changes in hemolymph concentrations of diuretic neuropeptides, reabsorptive activity of the hindgut, and/or contractile activity of the hindgut, given that our assays were performed on whole mosquitoes.

The molecular and functional data of the present study are consistent with a previous ultrastructural study of Malpighian tubules in adult female *Ae. taeniorhynchus* that found the apical brush-border membrane of principal cells to be characterized by reductions in the volume of (1) microvilli and (2) mitochondria within the microvilli, in the Malpighian tubules of 24 h BF mosquitoes compared to those of NBF mosquitoes (Bradley, Sauerman & Nayar, 1984). Thus, molecular, ultrastructural, and functional lines of data suggest that mosquito Malpighian tubules reduce their capacity for active transepithelial transport and diuresis within 24 h after a blood meal.

### Mechanisms of xenobiotic detoxification and excretion

In addition to salt and water balance, Malpighian tubules play key roles in the detoxification and excretion of xenobiotics (Beyenbach, Skaer & Dow, 2010; Dow & Davies, 2006; O’Donnell, 2009). Among the more notable mechanisms are cytochrome P450 monoxygenases (CYP450s), glutathione *S*-transferases (GSTs), and ATP-binding cassette (ABC) transporters, which respectively contribute to phase I, II, and III detoxification.

In the present study, we found that the Malpighian tubules of NBF mosquitoes expressed 64 transcripts encoding putative CYP450s, with representatives from the mitochondrial, CYP2, CYP3, and CYP4 clades (Table S10). In general, members of the mitochondrial and CYP2 clades are involved with ecdysteroid production and developmental regulation (e.g., Halloween genes), whereas the CYP3 and CYP4 clades are typically associated with xenobiotic detoxification and insecticide resistance (Feyereisen, 2006). As shown in Table S10, 40 of the CYP450 transcripts expressed in Malpighian tubules of NBF mosquitoes belonged to the CYP3 clade, while 13, 8, and 3 transcripts belonged to the CYP4, mitochondrial, and CYP2 clades, respectively. Moreover, relative abundances (RPKM values) of the CYP3 transcripts were generally greater than those of the other family members, suggesting potentially important detoxification roles of CYP3 members in the Malpighian tubules of NBF mosquitoes.

In addition to CYP450s, 26 transcripts encoding putative GSTs were present in the Malpighian tubules of NBF mosquitoes, with representative cytosolic (e.g., delta, epsilon, etc.) and microsomal GSTs (Table S10). The insect-specific delta and epsilon classes, which are associated with xenobiotic detoxification and insecticide resistance, were most highly represented with six and seven transcripts, respectively. Notably, two transcripts encoding Xi GSTs were also present; members of this class possess heme-binding activity and may contribute to heme detoxification after a blood meal (Lumjuan et al., 2007). Moreover, 29 transcripts encoding putative ABC transporters were detected in the Malpighian tubules of NBF mosquitoes, with representation from the A (6 transcripts), B (6 transcripts), C (7 transcripts), and G (10 transcripts) sub-groups (Table S10).

Altogether, the Malpighian tubules express over (1) 33% of the 186 CYP450 genes, (2) 80% of the 32 GST genes, and 40% of the 71 ABC transporter genes that have recently been identified in the genome of *Ae. albopictus* (Chen et al., 2015). Thus, the Malpighian tubules of NBF mosquitoes appear to have a high molecular capacity for phase I, II, and III detoxification of xenobiotics.

**Figure 4:**
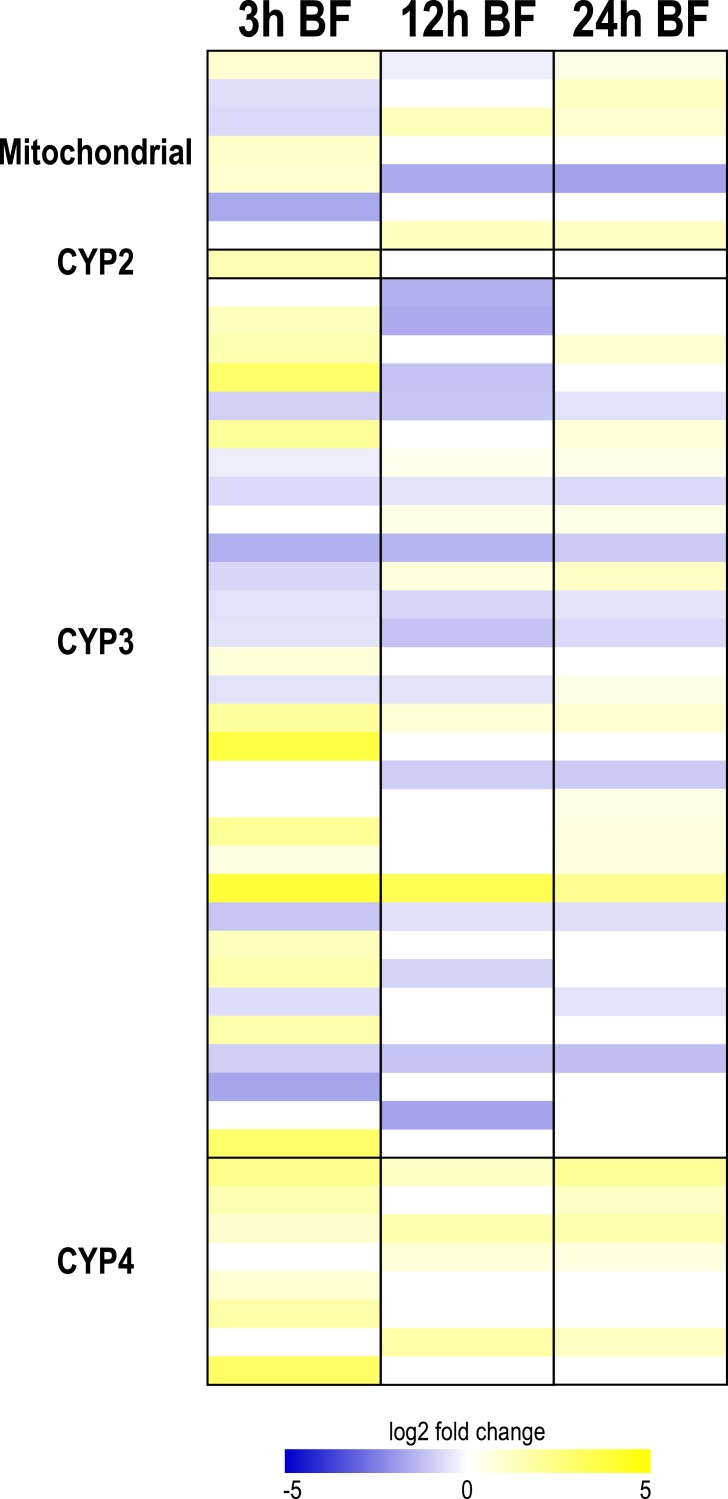
Heat map showing differential expression of transcripts encoding cytochrome P450 monoxygenases (CYP450s). Specific transcripts are listed in Table S11.

### Changes after blood feeding

#### CYP450s

At each time point after a blood meal, several CYP450 transcripts from each clade were differentially expressed, with the exception of the CYP2 clade (Fig. 4). A diverse pattern of differential expression occurred in the mitochondrial CYP450 clade. At 3 and 12 h, there were similar numbers of up and downregulated transcripts (3 up and 3 down at 3 h; 2 up and 2 down at 12 h), whereas at 24 h most were upregulated (4 up and 1 down) (Fig. 4, Table S11). Interestingly, all of the differentially-expressed mitochondrial CYP450 transcripts were transiently up or downregulated, and in some cases showed an upregulation followed by a downregulation or vice versa. In the CYP2 clade only one member was differentially-expressed; it was upregulated at 3 h (Fig. 4, Table S11).

The functional implications of such changes in transcript expression are uncertain. However, the general trends we found in the differential expression of CYP302A1, CYP314A1, and CYP15A1 (Table S11) were very similar to those observed in the ovaries of *Ae. aegypti* where their differential expression correlates with peak periods of ovarian ecdysteroid production (Sieglaff, Duncan & Brown, 2005). Thus, a putative role of the Malpighian tubules in mosquito ecdysteroid synthesis after a blood meal should be further investigated. Consistent with this notion, ecdysteroid-synthesis was detected in the abdomen of adult female *Ae. aegypti* after the ovaries were removed (Sieglaff, Duncan & Brown, 2005).

The CYP3 clade also showed a dynamic pattern of differential expression after a blood meal (Fig. 4). At 3 h and 24 h, a similar number of CYP3 transcripts were up- and downregulated (13 up and 12 down at 3 h; 11 up and 9 down at 24 h), whereas at 12 h, most CYP3 transcripts were downregulated (5 up and 14 down) (Fig. 4, Table S11). Only 2 CYP3 transcripts were upregulated at all 3 time points, whereas 7 CYP3 transcripts were downregulated at all 3 time points; moreover, several CYP3 transcripts were transiently up- or downregulated (Fig. 4, Table S11). Thus, the response of CYP3 transcripts to blood-feeding is complex and offers no universal trend. In contrast, transcripts in the CYP4 clade showed a concerted pattern of upregulation. A total of 6, 4, and 5 transcripts were upregulated at 3 h, 12 h, and 24 h, while none were downregulated; two of the CYP4 transcripts were upregulated at all 3 time points (Fig. 4, Table S11). These data suggest that members of the CYP4 clade may play important roles in the detoxification of blood meal metabolites.

Interestingly, several of the CYP3 and CYP4 transcripts that exhibited an upregulation at one or more time points after a blood meal in the present study have been implicated in metabolic resistance to insecticides in *Ae. aegypti* (Bariami et al., 2012; David et al., 2014; Dusfour et al., 2015; Reid et al., 2014; Vontas et al., 2012); i.e., CYP9J26, CYP9J28, CYP6BB2, CYP6M9, CYP6N9, CYP6N12, CYP6Z8, CYP6Z9, CYP9J9 v1, CYP9J10 v2, CYP9J19 v2, CYP4D24, CYP4H29, CYP4H30, CYP4H33 (Table S11). Also, CYP9M9, which is constitutively upregulated in Malpighian tubules after blood feeding (Table S11), has been shown to be upregulated in larvae of *Ae. aegypti* and *Ae. albopictus* reared in water containing toxic leaf litter (Kim & Muturi, 2012). Thus, the transcriptional regulation of the mosquito CYP3 and CYP4 members appears to respond to a broad variety of toxic insults from artificial or natural xenobiotics, including vertebrate blood.

#### GSTs

The cytosolic and microsomal GSTs showed a concerted upregulation after blood feeding (Table S11). At least 18 GST transcripts were upregulated at each time point, while only 2–3 transcripts were downregulated (Table S11). Moreover, 14 transcripts were upregulated at all 3 time points, in contrast to only one (i.e., GSTz1) that was constitutively downregulated (Table S11). Thus, the Malpighian tubules appear to increase their molecular capacity for GST-mediated detoxification after a blood meal, which is consistent with results of our earlier study (Esquivel, Cassone & Piermarini, 2014). Moreover, several GST transcripts that exhibited an upregulation at one or more time points after a blood meal have been implicated in metabolic resistance to insecticides in *Ae. aegypti* (Bariami et al., 2012; Dusfour et al., 2015; Vontas et al., 2012); i.e., GSTe2, GSTe4, GSTe7, GSTi1, GSTo1, and GSTx2 (Table S11). Thus, the transcriptional regulation of GSTs also appears to respond to a wide variety of xenobiotic stresses.

#### ABC transporters

In general, transcripts encoding ABC transporters were upregulated after blood feeding (Table S11). At 3 h and 24 h, most transcripts were upregulated (15 up, 8 down at 3 h; 11 up, 4 down at 24 h) (Table S11). At 12 h, similar numbers of transcripts were up- and downregulated (6 up, 7 down) (Table S11). Three transcripts were upregulated at all three time points, while two transcripts were downregulated at all three time points (Table S11). Thus, consistent with our previous study, the Malpighian tubules appear to increase their capacity for ABC transporter-mediated detoxification after a blood meal (Esquivel, Cassone & Piermarini, 2014). Only a few of the transcripts that were up-regulated after blood feeding have been associated with insecticide resistance in *Ae. aegypti*; i.e., AAEL005937 and AAEL008624 (Bariami et al., 2012; Vontas et al., 2012) (Table S11). Thus, the transcriptional response of ABC transporters to a blood meal appears unique to that found in insecticide-resistant mosquitoes, which may suggest that ABC transporters are more specific in the substrates they transport compared to substrates that are enzymatically modified by GSTs and CYP450s.

#### GST biochemical assay

**Figure 5:**
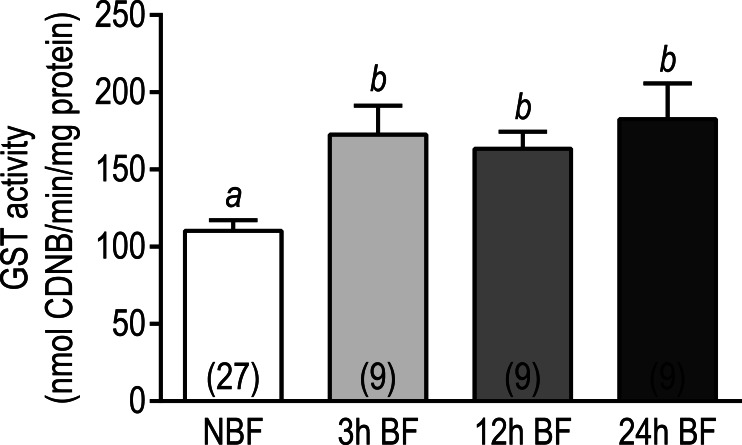
Effects of a blood meal on the GST activity in isolated Malpighian tubules of adult female *Ae. albopictus*. Values are means ± SEM, based on the number of independent trials in parentheses. Lower-case letters indicate statistical difference as determined by a one-way ANOVA with a Newman-Keuls posttest (*P* < 0.05).

Given that transcripts encoding GSTs in Malpighian tubules showed an overwhelming upregulation at each time point after blood feeding (Table S11), we sought to determine whether the biochemical activity of GST was affected. As shown in Fig. 5, the biochemical activity of GST significantly increased from 110.3 ± 6.8 nmol CDNB/min/mg protein in the Malpighian tubules of NBF mosquitoes to 172.6 ± 18.8 nmol CDNB/min/mg protein in the tubules of 3 h BF mosquitoes. The GST activity in the Malpighian tubules of 12 h and 24 h BF mosquitoes (12 h BF = 163.5 ± 11.1 nmol CDNB/min/mg protein; 24 h BF = 182.5 ± 23.1 nmol CDNB/min/mg protein) was significantly elevated compared to NBF controls and similar to the tubules of 3 h BF mosquitoes (Fig. 5). Thus, the upregulation of GST transcripts in Malpighian tubules 3–24 h after a blood meal correlates with an increase in biochemical GST activity by 3 h and persists through 24 h. These findings are consistent with results of recent studies that have found whole mosquito GST activity increases in *Ae. aegypti*, *C. pipiens*, and several anopheline species after one or more blood meal (Oliver & Brooke, 2014; Tripathy & Kar, 2015).

Taken together, the above transcriptomic and biochemical data suggest that the Malpighian tubules of mosquitoes enhance their capacity for xenobiotic metabolism and excretion within 24 h post-blood meal. These findings may in part explain why previous studies have found that the tolerance of mosquitoes to insecticides increases following a blood meal (Halliday & Feyereisen, 1987; Oliver & Brooke, 2014).

### Transcripts encoding enzymes involved with purine metabolism

In the majority of terrestrial insects studied to date, uric acid is the primary nitrogenous waste excreted (Dow, 2012). Uric acid is derived from amino acid and purine catabolism; it can be excreted with minimal water, is relatively non-toxic, and may also serve as a valuable antioxidant (Dow, 2012; Ramsey et al., 2010). In the Malpighian tubules of *D. melanogaster*, transcripts encoding enzymes associated with purine metabolism (e.g., xanthine dehydrogenase/*rosy*) are enriched (Chintapalli et al., 2013).

In the Malpighian tubules of NBF mosquitoes, we detected the expression of numerous transcripts associated with purine catabolism, including two that encode xanthine dehydrogenases, which catalyze the last step(s) of uric acid generation (enzyme codes ‘1.17.3.2’ and ‘1.17.1.4’ in Figs. S7, Table S12). In addition, several transcripts associated with enzymes that generate important precursors of uric acid (e.g., xanthine, hypoxanthine) derived from adenosine, D-ribose, guanosine, and xanthosine metabolism were detected (Figs. S7, Table S12). Thus, the Malpighian tubules of NBF mosquitoes appear to have a robust molecular capacity for purine metabolism and generating uric acid.

**Figure 6:**
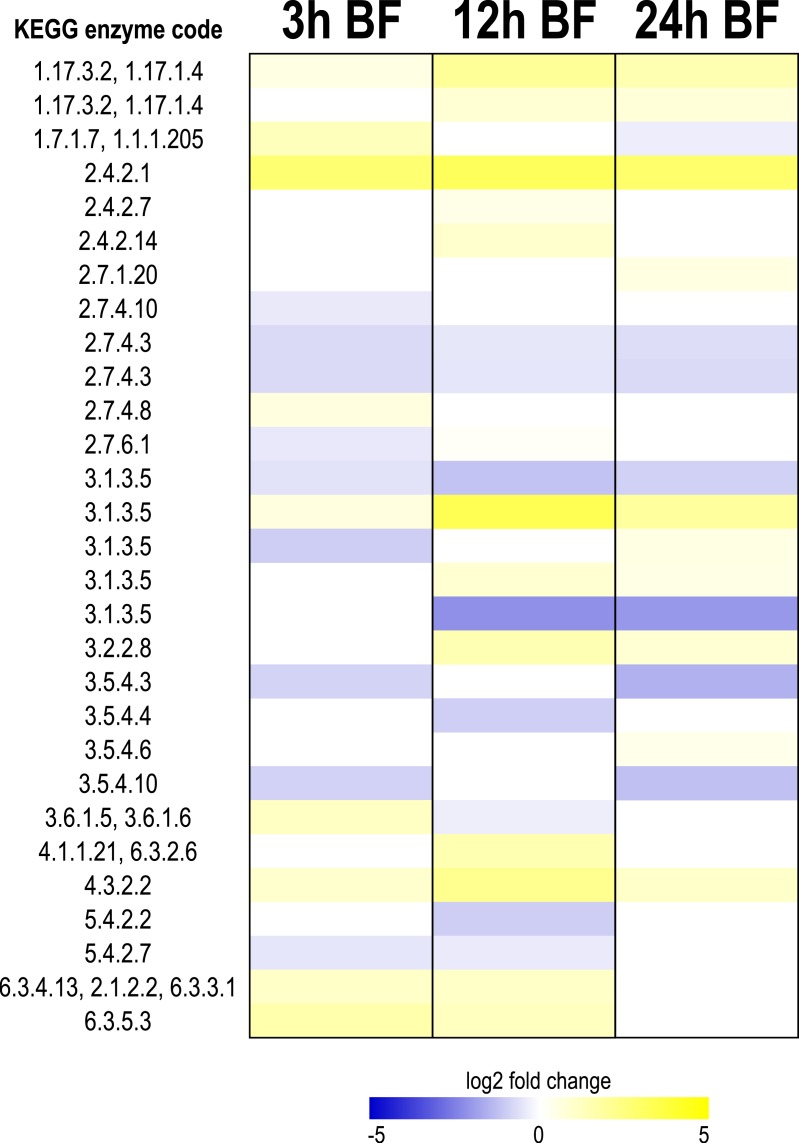
Heat map showing differential expression of transcripts encoding enzymes involved with purine metabolism. Specific transcripts for the KEGG enzyme codes are listed in Table S13. Figure S7 shows the positions and roles of the enzymes in the context of the KEGG metabolic pathway for purine metabolism.

### Changes after blood feeding

As shown in Fig. 6, after a blood meal, transcripts associated with purine metabolism showed a mixed response, but in general were upregulated. At each time point, the majority of transcripts were upregulated (10 up, 7 down at 3 h; 12 up, 6 down at 12 h; 11 up, 7 down at 24 h) (Fig. 6, Table S13). Four transcripts were upregulated at all 3 time points, whereas 3 were downregulated at all 3 time points (Fig. 6, Table S13). Among those upregulated at all 3 time points were transcripts encoding xanthine dehydrogenase (enzyme codes ‘1.17.3.2’ and ‘1.17.1.4’) and purine nucleoside phosphorylase (enzyme code ‘2.4.2.1’), which catalyze the most downstream steps of uric acid synthesis derived from inosine and xanthosine (Figs. S7, 6, Table S13). Notably, the pattern of xanthine dehydrogenase (XDH) transcript expression in Malpighian tubules following a blood meal is very similar to that observed for whole mosquito XDH biochemical activity in *Ae. aegypti*, which peaks ∼24 h after feeding (Von Dungern & Briegel, 2001).

#### Uric acid assay

**Figure 7:**
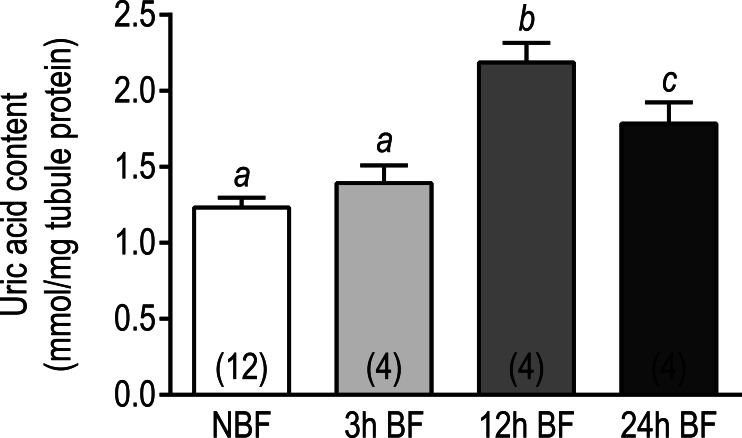
Effects of a blood meal on the uric acid content in isolated Malpighian tubules of adult female *Ae. albopictus*. Values are means ± SEM, based on the number of independent trials in parenthesis. Lower-case letters indicate statistical difference as determined by a one- way ANOVA with a Newman-Keuls posttest (*P* < 0.05).

We next sought to determine whether the above changes in transcript expression correlated with changes in the uric acid content of Malpighian tubules following a blood meal. As shown in Fig. 7, the uric acid content in the Malpighian tubules of 3 h BF mosquitoes (1.39 ± 0.12 mmol uric acid/mg) and NBF mosquitoes (1.23 ± 0.06 mmol uric acid/mg protein) were similar. However, in the Malpighian tubules of 12 h BF mosquitoes, the uric acid content significantly increased to 2.19 ± 0.13 mmol uric acid/mg protein (Fig. 7). The uric acid content in the Malpighian tubules of 24 h BF mosquitoes (1.785 ± 0.14 mmol uric acid/mg protein) was significantly greater than that in the tubules of NBF and 3 h BF mosquitoes, but significantly lower than that in the tubules of 12 h BF mosquitoes (Fig. 7).

Taken together, the above transcriptomic and biochemical data suggest that the Malpighian tubules of mosquitoes enhance their capacity to synthesize uric acid within 24 h post-blood meal. These findings are consistent with results from our previous study (Esquivel, Cassone & Piermarini, 2014) and correlate well with the peak rate of uric acid excretion found in *Ae. aegypti* mosquitoes after a blood meal, which occurs between 12 and 24 h after blood ingestion (Von Dungern & Briegel, 2001). Our data also suggest that the Malpighian tubules likely play a key role in the excretion of uric acid by mosquitoes following a blood meal.

### Conclusions and significance

The present study is the first to develop a *de novo* transcriptome of Malpighian tubules from NBF and BF mosquitoes and demonstrate that transcriptomic changes in mosquito Malpighian tubules after a blood meal are associated with functional changes to mosquitoes and the tubules. In particular, we find that within 3–24 h after a blood meal, the Malpighian tubules reinvest molecular resources from diuretic to detoxification mechanisms, which is correlated with at least (1) a lower diuretic capacity of the mosquitoes, (2) a higher biochemical capacity for GST-mediated phase II detoxification, and (3) a higher biochemical capacity for uric acid synthesis. The observed reinvestment of molecular resources is expected to better serve the physiological needs of the mosquito during this period of time. That is, the acute threat to mosquito salt and water balance associated with engorging on blood is alleviated by the post-prandial diuresis within 2 h after feeding (Williams, Hagedorn & Beyenbach, 1983). After this time, the energetically-expensive diuretic mechanisms would no longer be needed; instead, the mosquito must focus its resources on digesting blood proteins, detoxifying blood metabolites, and investing nutrients into their eggs for reproduction.

Our transcriptomic results also reveal the expression of transcripts encoding Na^+^∕Ca^2+^ exchangers, GPCRs, and putative transmembrane septate-junctional proteins that have not previously been described in mosquito Malpighian tubules. Elucidating the functional and/or regulatory roles of these mechanisms in Malpighian tubules will be critical to advancing our understanding of the basic physiology of mosquito Malpighian tubules and the physiological roles that Malpighian tubules play in mosquitoes. Furthermore, these mechanisms—and those that are upregulated after blood feeding—may serve as valuable molecular targets for disrupting the renal functions of mosquitoes with small molecules to facilitate the development of novel mosquitocides for combatting insecticide resistant mosquitoes (Beyenbach et al., 2015; Raphemot et al., 2013).

## Supplemental Information

10.7717/peerj.1784/supp-1 Supplemental Information 1 Supplemental Methods

10.7717/peerj.1784/supp-2 Table S1 Uncategorized DAVID functional clusters enriched among transcripts expressed in the Malpighian tubules of NBF mosquitoes See Table S2 for list of transcripts included in these clusters.

10.7717/peerj.1784/supp-3 Table S2 Transcripts within DAVID functional clusters listed in Tables 2 and S1 Each general functional category is a separate sheet.

10.7717/peerj.1784/supp-4 Table S3 Up- and down-regulated functional clusters within each general functional category Category names are highlighted gray in bold and italicized text. Each sheet represents a different time point (i.e., 3 h, 12 h, and 24 h). The transcripts associated with each functional cluster are found in Tables S4–S7.

10.7717/peerj.1784/supp-5 Table S4 Transcripts associated with the up- and down-regulated functional clusters in the Malpighian tubules of 3 h BF mosquitoes Each sheet represents a different functional category and direction of regulation.

10.7717/peerj.1784/supp-6 Table S5 Transcripts associated with the up- and down-regulated functional clusters in the Malpighian tubules of 12 h BF mosquitoes Each sheet represents a different functional category and direction of regulation.

10.7717/peerj.1784/supp-7 Table S6 Transcripts associated with the up- and down-regulated functional clusters in the Malpighian tubules of 24 h BF mosquitoes Each sheet represents a different functional category and direction of regulation.

10.7717/peerj.1784/supp-8 Table S7 Uncategorized transcripts that were up- or down-regulated Each sheet represents a different time point and direction of regulation.

10.7717/peerj.1784/supp-9 Table S8 Transcripts expressed in Malpighian tubules of NBF mosquitoes associated with active transepithelial fluid secretion/diuresis Transcripts encoding mechanisms of ion and water transport and ATP synthesis are found in Sheets 1 and 2, respectively.

10.7717/peerj.1784/supp-10 Table S9 List of transcripts and heat maps for transcripts differentially expressed in Malpighian tubules of BF mosquitoes associated with transepithelial fluid secretion/diuresis Transcripts encoding mechanisms of ion/water transport and ATP synthesis are found in Sheets 1 and 2, respectively. Transcripts encoding GPCRs and putative transmembrane septate junctional proteins are found in Sheets 3 and 4, respectively.

10.7717/peerj.1784/supp-11 Table S10 Transcripts expressed in Malpighian tubules of NBF mosquitoes associated with xenobiotic detoxification Transcripts encoding cytochrome P450 monoxygenases, glutathione S-transferases, and ABC transporters are found in Sheets 1, 2, and 3, respectively.

10.7717/peerj.1784/supp-12 Table S11 List of transcripts differentially expressed in Malpighian tubules of BF mosquitoes associated with detoxification Transcripts encoding cytochrome P450 monoxygenases (CYP450s), glutathione S-transferases (GSTs), and ABC transporters are found in Sheets 1, 2, and 3, respectively.

10.7717/peerj.1784/supp-13 Table S12 Transcripts expressed in Malpighian tubules of NBF mosquitoes associated with purine metabolism and their associated KEGG enzyme codes Sheet 1 is sorted by KEGG enzyme code (low to high). Sheet 2 is sorted by RPKM value (high to low). See Fig. S7 for the reactions catalyzed by each enzyme code.

10.7717/peerj.1784/supp-14 Table S13 List of transcripts differentially expressed in Malpighian tubules of BF mosquitoes associated with purine metabolism

10.7717/peerj.1784/supp-15 Figure S1 Venn diagram showing pair-wise comparisons (BLASTn) of the 8,047 *Ae. albopictus* transcripts with a significant ortholog (*E*-value 10^−6^) in the 4 dipteran transcriptomes queried The Venn diagram was generated using Venny 2.0 (Oliveros, 2007–2015).

10.7717/peerj.1784/supp-16 Figure S2 Distribution of contigs by length Contigs longer than 8,000 bp (*n* = 95) are not shown.

10.7717/peerj.1784/supp-17 Figure S3 Distribution of ortholog hit ratios for the annotated *Ae. albopictus* transcripts

10.7717/peerj.1784/supp-18 Figure S4 Venn diagram showing the relationships of differentially-expressed transcripts in the Malpighian tubules of BF mosquitoes In the 3 h BF tubules, over 50% of the differentially-expressed transcripts were unique to that time period, whereas in the 12 h and 24 h BF tubules, ∼40% of the differentially-expressed transcripts were unique to their respective period. ‘U’ and ‘D’ indicate numbers of up-regulated and down-regulated transcripts, respectively. BF, blood fed. The Venn diagram was generated using Venny 2.0 (Oliveros, 2007–2015).

10.7717/peerj.1784/supp-19 Figure S5 Molecular model of transepithelial fluid secretion in mosquito Malpighian tubules See text for details. Gap junctions (innexins) form connections between principal cells and between principal and stellate cells, where they may synchronize the epithelial cells and contribute to the metabolic regulation of fluid secretion (Beyenbach & Piermarini, 2011; Piermarini & Calkins, 2014). The receptors for DH_31_ and kinin are G protein-coupled receptors (GPCRs) that localize to the basolateral membranes of principal and stellate cells, respectively (Halberg et al., 2015; Kersch & Pietrantonio, 2011; Kwon et al., 2012; Lu, Kersch & Pietrantonio, 2011); transcripts encoding DH_44_ GPCRs are expressed in the Malpighian tubules of *Ae. aegypti* and *An. gambiae*, but have yet to be localized (Jagge & Pietrantonio, 2008; Overend et al., 2015). AE, anion exchanger; AQP, aquaporin; ATP, adenosine triphosphate; DH, diuretic hormone; KCC, K,Cl cotransporter; Kir, inward rectifier K^+^ channel; NDAE, Na-driven anion exchanger; NHA, Na/H antiporter; NHE, Na/H exchanger; NKA, Na, K-ATPase; NKCC, Na,K,Cl cotransporter; SJ, septate junction.

10.7717/peerj.1784/supp-20 Figure S6 Schematic of functional/metabolic coupling between a mitochondrion and the *V*-type H^+^-ATPase in an apical microvillus of the principal cell brush border Adenosine triphosphate (ATP) that is generated by the mitochondrion is shuttled to the *V*-type H^+^-ATPase and provides energy for the translocation of H^+^ across the apical membrane. The resulting adenosine diphosphate (ADP) is recycled by the mitochondrion. Redrawn and modified from Beyenbach (2001). NHA,Na/H antiporter.

10.7717/peerj.1784/supp-21 Figure S7 Simplified KEGG pathway of purine metabolism, resulting in uric acid production Numbers indicate KEGG enzyme codes corresponding to transcripts that were expressed in Malpighian tubules of NBF mosquitoes (see Table S12 for transcripts that correspond to enzyme codes). Dashed lines indicate potential enzymatic reactions for which no corresponding transcripts were detected in the transcriptome. AMP, Adenosine monophosphate; ADP, adenosine diphosphate; dAMP; deoxyadenosine monophosphate; GMP, guanosine monophosphate; GDP, guanosine diphosphate; dGMP, deoxyguanosine monophosphate; IMP, inosine monophosphate; XMP, xanthosine monophosphate. Redrawn and modified from Ramsey et al. (2010).

10.7717/peerj.1784/supp-22 Supplemental Information 2 Raw sequencing data of assembled transcripts from Malpighian tubules of non-blood fed and blood fed adult female *Aedes albopictus* mosquitoes

10.7717/peerj.1784/supp-23 Supplemental Information 3 RPKM values for all transcripts

10.7717/peerj.1784/supp-24 Supplemental Information 4 Raw data for functional assays

## Additional Information and Declarations

### Data Availability

The following information was supplied regarding data availability: The paired-end reads can be retrieved at the SRA (Sequence Read Archive) of the NCBI (National Center for Biotechnology Information) under the Bioproject accession number PRJNA279095(experiment SRX964103). This *de novo* transcriptome was deposited into the NCBI Transcriptome Shotgun Assembly (TSA) database under BioSample number SRS883933and accession number GCZT00000000.

## References

[ref-2] Araújo AR, Reis M, Rocha H, Aguiar B, Morales-Hojas R, Macedo-Ribeiro S, Fonseca NA, Reboiro-Jato D, Reboiro-Jato M, Fdez-Riverola F, Vieira CP, Vieira J (2013). The *Drosophila melanogaster methuselah* gene: a novel gene with ancient functions. PLoS ONE.

[ref-3] Bariami V, Jones CM, Poupardin R, Vontas J, Ranson H (2012). Gene amplification, ABC transporters and cytochrome P450s: unraveling the molecular basis of pyrethroid resistance in the dengue vector, *Aedes aegypti*. PLoS Neglected Tropical Diseases.

[ref-6] Beyenbach KW (2001). Energizing epithelial transport with the vacuolar H^+^-ATPase. News in Physiological Sciences.

[ref-4] Beyenbach KW (2003). Transport mechanisms of diuresis in Malpighian tubules of insects. Journal of Experimental Biology.

[ref-5] Beyenbach KW (2012). A dynamic paracellular pathway serves diuresis in mosquito Malpighian tubules. Annals of the N Y Academy of Sciences.

[ref-7] Beyenbach KW, Pannabecker TL, Nagel W (2000). Central role of the apical membrane H^+^-ATPase in electrogenesis and epithelial transport in Malpighian tubules. Journal of Experimental Biology.

[ref-8] Beyenbach KW, Petzel DH (1987). Diuresis in mosquitoes role of a natriuretic factor. News in Physiological Sciences.

[ref-10] Beyenbach KW, Piermarini PM, Evans DH (2009). Osmotic and ionic regulation in insects. Osmotic and ionic regulation: cells and animals.

[ref-9] Beyenbach KW, Piermarini PM (2011). Transcellular and paracellular pathways of transepithelial fluid secretion in Malpighian (renal) tubules of the yellow fever mosquito *Aedes aegypti*. Acta Physiologica.

[ref-11] Beyenbach KW, Skaer H, Dow JA (2010). The developmental, molecular, and transport biology of Malpighian tubules. Annual Review of Entomology.

[ref-12] Beyenbach KW, Yu Y, Piermarini PM, Denton J (2015). Targeting renal epithelial channels for the control of insect vectors. Tissue Barriers.

[ref-13] Bradley TJ, Sauerman DMJ, Nayar JK (1984). Early cellular responses in the Malpighian tubules of the mosquito *Aedes taeniorhynchus* to infection with *Dirofilaria immitis* Nematoda. Journal of Parasitology.

[ref-14] Briegel H (1986). Protein catabolism and nitrogen partitioning during oögenesis in the mosquito *Aedes aegypti*. Journal of Insect Physiology.

[ref-15] Brogdon WG, Barber AM (1990). Microplate assay of glutathione *S*-transferase activity for resistance detection in single-mosquito triturates. Comparative Biochemistry and Physiology Part B: Comparative Biochemistry.

[ref-16] Caers J, Verlinden H, Zels S, Vandersmissen HP, Vuerinckx K, Schoofs L (2012). More than two decades of research on insect neuropeptide GPCRs: an overview. Frontiers in Endocrinology.

[ref-18] Chen X-G, Jiang X, Gu J, Xu M, Wu Y, Deng Y, Zhang C, Bonizzoni M, Dermauw W, Vontas J, Armbruster P, Huang X, Yang Y, Zhang H, He W, Peng H, Liu Y, Wu K, Chen J, Lirakis M, Topalis P, Van Leeuwen T, Hall AB, Jiang X, Thorpe C, Mueller RL, Sun C, Waterhouse RM, Yan G, Tu ZJ, Fang X, James AA (2015). Genome sequence of the Asian Tiger mosquito, *Aedes albopictus*, reveals insights into its biology, genetics, and evolution. Proceedings of the National Academy of Sciences.

[ref-19] Chintapalli V, Wang J, Herzyk P, Davies S, Dow J (2013). Data-mining the FlyAtlas online resource to identify core functional motifs across transporting epithelia. BMC Genomics.

[ref-21] Coast G (2007). The endocrine control of salt balance in insects. General and Comparative Endocrinology.

[ref-20] Coast GM (2009). Neuroendocrine control of ionic homeostasis in blood-sucking insects. Journal of Experimental Biology.

[ref-23] Crawford JE, Guelbeogo WM, Sanou A, Traoré A, Vernick KD, Sagnon NF, Lazzaro BP (2010). *De novo* transcriptome sequencing in *Anopheles funestus* using Illumina RNA-Seq technology. PLoS ONE.

[ref-24] David JP, Faucon F, Chandor-Proust A, Poupardin R, Riaz MA, Bonin A, Navratil V, Reynaud S (2014). Comparative analysis of response to selection with three insecticides in the dengue mosquito *Aedes aegypti* using mRNA sequencing. BMC Genomics.

[ref-25] Dow JAT, Simpson SJ, Douglas AE (2012). Excretion and salt and water regulation. The insects.

[ref-26] Dow JA, Davies SA (2006). The Malpighian tubule: rapid insights from post-genomic biology. Journal of Insect Physiology.

[ref-27] Dusfour I, Zorrilla P, Guidez A, Issaly J, Girod R, Guillaumot L, Robello C, Strode C (2015). Deltamethrin resistance mechanisms in *Aedes aegypti p*opulations from three French overseas territories worldwide. PLoS Neglected Tropical Diseases.

[ref-28] Esquivel CJ, Cassone BJ, Piermarini PM (2014). Transcriptomic evidence for a dramatic functional transition of the Malpighian tubules after a blood meal in the Asian tiger mosquito *Aedes albopictus*. PLoS Neglected Tropical Diseases.

[ref-29] Ewen-Campen B, Shaner N, Panfilio KA, Suzuki Y, Roth S, Extavour CG (2011). The maternal and early embryonic transcriptome of the milkweed bug *Oncopeltus fasciatus*. BMC Genomics.

[ref-30] Feyereisen R (2006). Evolution of insect P450. Biochemical Society Transactions.

[ref-32] Gentleman R, Carey V, Bates D, Bolstad B, Dettling M, Dudoit S, Ellis B, Gautier L, Ge Y, Gentry J, Hornik K, Hothorn T, Huber W, Iacus S, Irizarry R, Leisch F, Li C, Maechler M, Rossini A, Sawitzki G, Smith C, Smyth G, Tierney L, Yang J, Zhang J (2004). Bioconductor: open software development for computational biology and bioinformatics. Genome Biology.

[ref-33] Habig WH, Pabst MJ, Jakoby WB (1974). Glutathione *S*-transferases: the first enzymatic step in mercapturic acid formation. Journal of Biological Chemistry.

[ref-35] Halliday WR, Feyereisen R (1987). Why does DDT toxicity change after a blood meal in adult female *Culex pipiens*?. Pesticide Biochemistry and Physiology.

[ref-36] Hanlon CD, Andrew DJ (2015). Outside-in signaling—a brief review of GPCR signaling with a focus on the *Drosophila* GPCR family. Journal of Cell Science.

[ref-37] Hemingway J (1998). Techniques to detect insecticide resistance mechanisms (field and laboratory manual).

[ref-38] Hine RM, Rouhier MF, Park ST, Qi Z, Piermarini PM, Beyenbach KW (2014). The excretion of NaCl and KCl loads in mosquitoes: 1. Control data. American Journal of Physiology Regulatory Integrative and Comparative Physiology.

[ref-39] Huang da W, Sherman BT, Lempicki RA (2009). Systematic and integrative analysis of large gene lists using DAVID bioinformatics resources. Nature Protocols.

[ref-40] Huang X, Poelchau MF, Armbruster PA (2015). Global transcriptional dynamics of diapause induction in non-blood-fed and blood-fed *Aedes albopictus*. PLoS Neglected Tropical Diseases.

[ref-41] Izumi Y, Furuse M (2014). Molecular organization and function of invertebrate occluding junctions. Seminars in Cell & Developmental Biology.

[ref-42] Izumi Y, Yanagihashi Y, Furuse M (2012). A novel protein complex, Mesh–Ssk, is required for septate junction formation in the Drosophila midgut. Journal of Cell Science.

[ref-43] Jagge CL, Pietrantonio PV (2008). Diuretic hormone 44 receptor in Malpighian tubules of the mosquito *Aedes aegypti:* evidence for transcriptional regulation paralleling urination. Insect Molecular Biology.

[ref-44] Johard HAD, Coast GM, Mordue W, Nässel DR (2003). Diuretic action of the peptide locustatachykinin I: cellular localisation and effects on fluid secretion in Malpighian tubules of locusts. Peptides.

[ref-47] Khananshvili D (2013). The SLC8 gene family of sodium–calcium exchangers (NCX)—structure, function, and regulation in health and disease. Molecular Aspects of Medicine.

[ref-48] Kim CH, Muturi EJ (2012). Relationship between leaf litter identity, expression of cytochrome P450 genes and life history traits of *Aedes aegypti* and *Aedes albopictus*. Acta Tropica.

[ref-50] Li H, Durbin R (2009). Fast and accurate short read alignment with Burrows-Wheeler transform. Bioinformatics.

[ref-53] Love MI, Huber W, Anders S (2014). Moderated estimation of fold change and dispersion for RNA-Seq data with DESeq2. Genome Biology.

[ref-54] Lu HL, Kersch C, Pietrantonio PV (2011). The kinin receptor is expressed in the Malpighian tubule stellate cells in the mosquito *Aedes aegypti* (L.): a new model needed to explain ion transport?. Insect Biochemistry and Molecular Biology.

[ref-55] Lumjuan N, Stevenson BJ, Prapanthadara LA, Somboon P, Brophy PM, Loftus BJ, Severson DW, Ranson H (2007). The *Aedes aegypti* glutathione transferase family. Insect Biochemistry and Molecular Biology.

[ref-59] O’Donnell MJ (2009). Too much of a good thing: how insects cope with excess ions or toxins in the diet. Journal of Experimental Biology.

[ref-60] Oliver SV, Brooke BD (2014). The effect of multiple blood-feeding on the longevity and insecticide resistant phenotype in the major malaria vector *Anopheles arabiensis* (Diptera: Culicidae). Parasites and Vectors.

[ref-62] O’Neil ST, Dzurisin JD, Carmichael RD, Lobo NF, Emrich SJ, Hellmann JJ (2010). Population-level transcriptome sequencing of nonmodel organisms *Erynnis propertius* and *Papilio zelicaon*. BMC Genomics.

[ref-63] O’Neil S, Emrich S (2013). Assessing *De Novo* transcriptome assembly metrics for consistency and utility. BMC Genomics.

[ref-64] Overend G, Cabrero P, Halberg KA, Ranford-Cartwright LC, Woods DJ, Davies SA, Dow JAT (2015). A comprehensive transcriptomic view of renal function in the malaria vector, *Anopheles gambiae*. Insect Biochemistry and Molecular Biology.

[ref-65] Padrón A, Molina-Cruz A, Quinones M, Ribeiro JM, Ramphul U, Rodrigues J, Shen K, Haile A, Ramirez JL, Barillas-Mury C (2014). In depth annotation of the *Anopheles gambiae* mosquito midgut transcriptome. BMC Genomics.

[ref-66] Patrick ML, Aimanova K, Sanders HR, Gill SS (2006). *P*-type Na^+^∕K^+^-ATPase and *V*-type H^+^-ATPase expression patterns in the osmoregulatory organs of larval and adult mosquito *Aedes aegypti*. Journal of Experimental Biology.

[ref-67] Petzel DH, Hagedorn HH, Beyenbach KW (1985). Preliminary isolation of mosquito natriuretic factor. American Journal of Physiology.

[ref-68] Petzel DH, Hagedorn HH, Beyenbach KW (1986). Peptide nature of two mosquito natriuretic factors. American Journal of Physiology.

[ref-70] Piermarini P, Gillen C, Hyndman KA, Pannabecker TL (2015). Non-traditional models: the molecular physiology of sodium and water transport in mosquito Malpighian tubules. Sodium and water homeostasis.

[ref-71] Pietrantonio PV, Jagge C, Taneja-Bageshwar S, Nachman RJ, Barhoumi R (2005). The mosquito *Aedes aegypti* (L.) leucokinin receptor is a multiligand receptor for the three *Aedes* kinins. Insect Molecular Biology.

[ref-72] Poelchau MF, Reynolds JA, Denlinger DL, Elsik CG, Armbruster PA (2011). A *de novo* transcriptome of the Asian tiger mosquito, *Aedes albopictus*, to identify candidate transcripts for diapause preparation. BMC Genomics.

[ref-74] Ramsey JS, MacDonald SJ, Jander G, Nakabachi A, Thomas GH, Douglas AE (2010). Genomic evidence for complementary purine metabolism in the pea aphid, *Acyrthosiphon pisum*, and its symbiotic bacterium *Buchnera aphidicola*. Insect Molecular Biology.

[ref-75] Raphemot R, Rouhier MF, Hopkins CR, Gogliotti RD, Lovell KM, Hine RM, Ghosalkar D, Longo A, Beyenbach KW, Denton JS, Piermarini PM (2013). Eliciting renal failure in mosquitoes with a small-molecule inhibitor of inward-rectifying potassium channels. PLoS ONE.

[ref-77] Reid WR, Thornton A, Pridgeon JW, Becnel JJ, Tang F, Estep A, Clark GG, Allan S, Liu N (2014). Transcriptional analysis of four family 4 P450s in a Puerto Rico strain of *Aedes aegypti* (Diptera: Culicidae) compared with an Orlando strain and their possible functional roles in permethrin resistance. Journal of Medical Entomology.

[ref-76] Reidenbach KR, Cook S, Bertone MA, Harbach RE, Wiegmann BM, Besansky NJ (2009). Phylogenetic analysis and temporal diversification of mosquitoes (Diptera: Culicidae) based on nuclear genes and morphology. BMC Evolutionary Biology.

[ref-78] Sawyer DB, Beyenbach KW (1985). Dibutyryl-cAMP increases basolateral sodium conductance of mosquito Malpighian tubules. American Journal of Physiology Regulatory Integrative and Comparative Physiology.

[ref-79] Schnetkamp PPM (2013). The SLC24 gene family of Na^+^∕Ca^2+^–K^+^ exchangers: from sight and smell to memory consolidation and skin pigmentation. Molecular Aspects of Medicine.

[ref-81] Sieglaff DH, Duncan KA, Brown MR (2005). Expression of genes encoding proteins involved in ecdysteroidogenesis in the female mosquito, *Aedes aegypti*. Insect Biochemistry and Molecular Biology.

[ref-82] Skaer NJV, Nässel DR, Maddrell SHP, Tublitz NJ (2002). Neurochemical fine tuning of a peripheral tissue: peptidergic and aminergic regulation of fluid secretion by Malpighian tubules in the tobacco hawkmoth *M. sexta*. Journal of Experimental Biology.

[ref-83] Tiburcy F, Beyenbach KW, Wieczorek H (2013). Protein kinase A-dependent and -independent activation of the V-ATPase in Malpighian tubules of *Aedes aegypti*. Journal of Experimental Biology.

[ref-84] Tripathy A, Kar SK (2015). Feeding stage, species, body part and sex-specific activity of glutathione S-transferase in mosquito. Tropical Biomedicine.

[ref-85] Van Belleghem SM, Roelofs D, Van Houdt J, Hendrickx F (2012). *De novo* transcriptome assembly and SNP discovery in the wing polymorphic salt marsh beetle *Pogonus chalceus* (Coleoptera, Carabidae). PLoS ONE.

[ref-86] Von Dungern P, Briegel H (2001). Enzymatic analysis of uricotelic protein catabolism in the mosquito Aedes aegypti. Journal of Insect Physiology.

[ref-87] Vontas J, Kioulos E, Pavlidi N, Morou E, Della Torre A, Ranson H (2012). Insecticide resistance in the major dengue vectors *Aedes albopictus* and *Aedes aegypti*. Pesticide Biochemistry and Physiology.

[ref-88] Weng XH, Huss M, Wieczorek H, Beyenbach KW (2003). The *V*-type H^+^-ATPase in Malpighian tubules of *Aedes aegypti*: localization and activity. Journal of Experimental Biology.

[ref-89] Wiegmann BM, Trautwein MD, Winkler IS, Barr NB, Kim JW, Lambkin C, Bertone MA, Cassel BK, Bayless KM, Heimberg AM, Wheeler BM, Peterson KJ, Pape T, Sinclair BJ, Skevington JH, Blagoderov V, Caravas J, Kutty SN, Schmidt-Ott U, Kampmeier GE, Thompson FC, Grimaldi DA, Beckenbach AT, Courtney GW, Friedrich M, Meier R, Yeates DK (2011). Episodic radiations in the fly tree of life. Proceedings of the National Academy of Sciences of the United States of America.

[ref-90] Williams JC, Hagedorn HH, Beyenbach KW (1983). Dynamic changes in flow rate and composition of urine during the post blood meal diuresis in *Aedes aegypti*. Journal of Comparative Physiology A.

[ref-91] Yanagihashi Y, Usui T, Izumi Y, Yonemura S, Sumida M, Tsukita S, Uemura T, Furuse M (2012). Snakeskin, a membrane protein associated with smooth septate junctions, is required for intestinal barrier function in *Drosophila*. Journal of Cell Science.

[ref-92] Yu MJ, Beyenbach KW (2002). Leucokinin activates Ca^2+^-dependent signal pathway in principal cells of *Aedes aegypti* Malpighian tubules. American Journal of Physiology Renal Physiology.

[ref-93] Zhu G, Zhong D, Cao J, Zhou H, Li J, Liu Y, Bai L, Xu S, Wang M-H, Zhou G, Chang X, Gao Q, Yan G (2014). Transcriptome profiling of pyrethroid resistant and susceptible mosquitoes in the malaria vector, *Anopheles sinensis*. BMC Genomics.

