# Supplementary material for: A de novo transcriptome of the Malpighian tubules in non-blood-fed and blood-fed Asian tiger mosquitoes Aedes albopictus: insights into diuresis, detoxification, and blood meal processing
Source: PeerJ. 2016 Mar 10;4:e1784. doi: 10.7717/peerj.1784 (PMC4793337; doi:10.7717/peerj.1784)
Supplement: Supplemental Information 1 [file peerj-04-1784-s001.doc]

**Supplemental Methods**

*Trimming and filtering data.*

The sequencing quality of the 21 cDNA libraries was assessed using ‘FASTQC’ tool version 0.10.1 (Andrews, 2010). Barcoded-adapters were removed with ‘CUTADAPT’ tool version 1.2.1 (Martin, 2011) with an error rate of 0.1 and a minimum overlap length of 6. Short (< 20 bp), low quality (< 20 Phred score), and broken (unbalanced) reads were trimmed with ‘Trim the reads’ tool, version 1.2.2. Further quality assessment (e.g., principal component analysis) and filtering of contigs were performed following established bioinformatics protocols (Cassone et al., 2014; Crawford et al., 2010; Poelchau et al., 2011; Poelchau et al., 2013).

*Transcriptome reference-free assembly.*

We performed the *de novo* assembly on 7 trimmed, paired-end cDNA libraries (4 NBF libraries and 3 BF libraries, 1 per time point; ~115 million trimmed-reads total); use of all 21 cDNA libraries was not possible due to computational limitations. The libraries were randomized and combined using ‘FASTQ/A shuffle’ tool version 1.0.0 (Andrews, 2010). The assembly was executed with the ‘Rnnotator automated pipeline’ (Martin et al., 2010) set with the following optimized parameters: ‘coverage cutoff when splitting contigs’ = 3; ‘rare kmer length’ = 24; ‘rare kmer count’ < 3; ‘count minimum contigs length’ = 100; ‘precursor threshold’ = 0.1; ‘overlap length for clustering and extension’ = 200; and ‘BWA’ for the alignment of stranded reads. Low quality, low complexity duplicates, and rare k-mers were filtered. The assembler used was ‘OASES’ version 0.2.08 (Schulz et al., 2012), setting the kmer sizes at 53, 59, 65, 71, 77, 83, 89 and 95. Redundant contigs were removed with ‘CD-HIT-EST’ version 4.6.1 (Fu et al., 2012; Li & Godzik, 2006), set with a ‘word size filter’ of n = 8 (threshold 0.9 ~ 1) and ≥ 95% similarity, resulting in 28,189 contigs.

*Transcriptomic reference-based filtering.*

The trimmed reads from the 21 cDNA libraries were mapped onto the 28,189 contigs obtained from CD-HIT-EST (see above) using the ‘Burrow-Wheeler Aligner’ version 1.2.3 (Li & Durbin, 2009). If a contig within a NBF or BF sample had ≤ 10 mapped reads in any of its replicates then it was removed. This resulted in 22,802 contigs that were subsequently submitted to a pair-wise BLASTn comparison (*E*-value value ≤ 10^-6^;version 0.100 MCIC-Galaxy pipeline) using the transcriptome of the closely related mosquito *Aedes aegypti* ([www.vectorbase.org](http://www.vectorbase.org/), Liverpool strain, Aaegl3.1, v1.00, 18,383 sequences); the recently sequenced transcriptome of *Ae. albopictus* was unavailable at the time of our annotation (Chen et al., 2015). The XML file from the BLASTn search was managed with the Java^TM^-platform-based BLAST2GO software version 2.7.2 (Conesa et al., 2005) to retrieve the best hit using the ‘Top Blast’ tool. Contigs that did not significantly match an *Ae. aegypti* ortholog were submitted under the same parameters to a BLASTn search with the following transcriptomes until a significant match was made: *Culex quinquefasciatus* ([www.vectorbase.org](http://www.vectorbase.org/), Johannesburg strain predicted transcript sequences, CpipJ2.1, v1.00, 19,427 sequences); *An. gambiae* ([www.vectorbase.org](http://www.vectorbase.org/), Pest Strain AgamP4.1 v1.00, 15,478 sequences), and *Drosophila melanogaster* ([www.flybase.org](http://www.flybase.org/), dmell-all-transcripts-r5.57,vFB2014_03, released May 9, 2014, 30,306 sequences). Contigs that did not significantly match an ortholog in any of the above dipteran transcriptomes were submitted to a final BLASTn using the NCBI non-redundant transcriptomic database (release 65, May 12, 2014). If more than one contig associated with a particular ortholog in the BLASTn results, then the corresponding contigs were consolidated and the one with the longest length was retained. The above analyses resulted in ortholog validation of 8,232 non-redundant contigs or transcripts, which we refer to as ‘*Ae. albopictus* transcripts’.

*Transcript annotation.*

A pair-wise comparison approach described previously by Cassone et al. (2014) was used to find the dipteran transcriptome (among *Ae. aegypti, An. gambiae, C. quinquefasciatus,* and *D. melanogaster*) that annotated the largest number of unique *Ae. albopictus* transcripts and use it as the main reference. To annotate the transcripts, a BLASTx (version 1.00, MCIC-Galaxy pipeline) analysis was performed in a similar fashion as that described for the BLASTn in ‘*Transcriptomic reference-based filtering’.* In brief, the XML files from the BLASTx were retrieved and merged with the Java^TM^-platform-based BLAST2GO software (Conesa et al., 2005), using the following parameters: ‘HPS length cutoff’ = 33; ‘position’ = 5; and ‘number of BLAST hits’ = 20. Mapping (to retrieve GO terms) and annotation (to assign functional terms) were executed using the following parameters: ‘*E*-value’ of 10^-6^, ‘annotation cutoff’ = 55; ‘GO weight’ = 5; and ‘Hps-hit coverage cutoff’ = 30. The predictions of protein domains and sites for the *Ae. albopictus* transcripts were performed with ‘InterProScan’ version 5.0 (Jones et al., 2014) via Java^TM^-platform-based BLAST2GO. Most of the *Ae. albopictus* transcripts (n = 7,731) were annotated with the *Ae. aegypti* database, followed by the *C. quinquefasciatus* (n = 239), *An. gambiae* (n = 67), and *D. melanogaster* (n = 10) databases. The remaining 185 transcripts were annotated with the NCBI nr database.

Altogether, the annotated transcripts comprise the adult female *Ae. albopictus* Malpighian tubule transcriptome. This *de novo* transcriptome was deposited into the NCBI Transcriptome Shotgun Assembly (TSA) database under BioSample number SRS883933 and accession number GCZT00000000.

*Functional clustering analysis of transcripts.*

In brief, the *Ae. albopictus* transcripts were first transformed to their respective *An. gambiae* ortholog ([www.vectorbase.org](http://www.vectorbase.org/); Pest Strain AgamP4.1 v1.00, 15,478 sequences) using the standalone tBLASTx (*E*-value < 10^-6^, max target seqs = 1, best hit score edge and best hit overhang = 0.2); *An. gambiae* is the only mosquito species for which a reference database is available in DAVID. The resulting XML file was imported to BLAST2GO to retrieve the best ortholog hit using the tool ‘Top-Blast.’ The *An. gambiae* transcript IDs were uploaded to DAVID and the functional annotation clustering was performed with the following parameters: ‘Similar Term Overlap’ = 3; ‘Similar Threshold’ = 0.5; ‘Initial Group Membership’ = 3; ‘Final Group Membership’ = 3; and ‘Multiple Linkage Threshold’ = 0.5. The DAVID functional clustering analysis cannot be performed on more than 3,500 transcripts at a time; thus, for the NBF samples, we performed it on two separate groups of transcripts (n = 3,101 and 3,104, respectively, sorted alphanumerically by *An. gambiae* ID). The DAVID analysis reported 1) clusters of transcripts functionally related, and 2) an enrichment score assigned to each cluster.
