# Supplementary material for: A de novo transcriptome of the Malpighian tubules in non-blood-fed and blood-fed Asian tiger mosquitoes Aedes albopictus: insights into diuresis, detoxification, and blood meal processing
Source: PeerJ. 2016 Mar 10;4:e1784. doi: 10.7717/peerj.1784 (PMC4793337; doi:10.7717/peerj.1784)
Supplement: Table S1 — See Table S2 for list of transcripts included in these clusters. [file peerj-04-1784-s002.doc]

**Table S1.** Uncategorized DAVID functional clusters enriched among transcripts expressed in the Malpighian tubules of NBF mosquitoes. See Table S2 for list of transcripts included in these clusters.

| **Functional cluster** | **Enrichment score** |
| --- | --- |
| WD40 | 150.97 |
| Nucleotide binding | 4.33 |
| Cofactor metabolic process | 3.69 |
| Zinc finger, FYVE-type | 2.44 |
| Cis-trans isomerase activity | 2.03 |
| N-acyltransferase activity | 2 |
| Axogenesis | 1.87 |
| Cofactor binding | 1.74 |
| Tetratricopeptide repeat | 1.55 |
| Developmental growth | 1.38 |
| Triglyceride metabolic process | 1.31 |
| Zinc-finger, RING-type | 1.3 |
