# Supplementary material for: A de novo transcriptome of the Malpighian tubules in non-blood-fed and blood-fed Asian tiger mosquitoes Aedes albopictus: insights into diuresis, detoxification, and blood meal processing
Source: PeerJ. 2016 Mar 10;4:e1784. doi: 10.7717/peerj.1784 (PMC4793337; doi:10.7717/peerj.1784)
Supplement: Figure S1 — The Venn diagram was generated using Venny 2.0 (Oliveros, 2007–2015). [file peerj-04-1784-s015.doc]

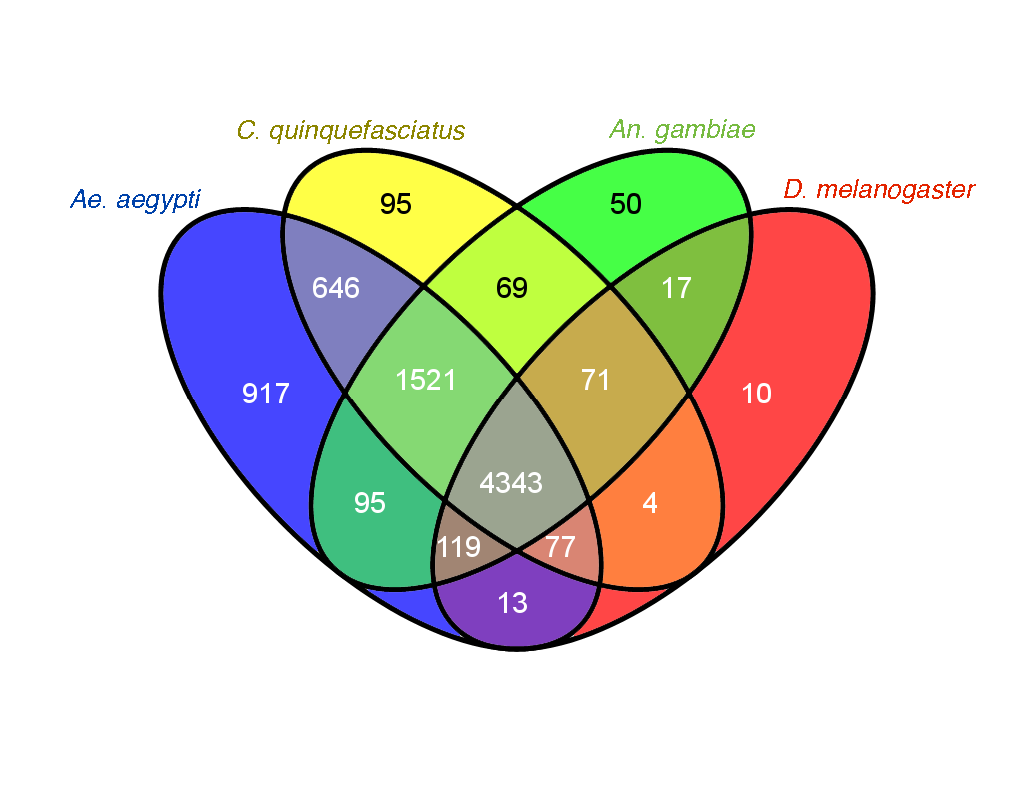


Figure S1. Venn diagram showing pair-wise comparisons (BLASTn) of the 8,047 *Ae. albopictus* transcripts with a significant ortholog (*E*-value 10^-6^) in the 4 dipteran transcriptomes queried*.* The Venn diagram was generated using Venny 2.0 (Oliveros, 2007-2015).
