# Supplementary figures and images for: A de novo transcriptome of the Malpighian tubules in non-blood-fed and blood-fed Asian tiger mosquitoes Aedes albopictus: insights into diuresis, detoxification, and blood meal processing
Source: PeerJ. 2016 Mar 10;4:e1784. doi: 10.7717/peerj.1784 (PMC4793337; doi:10.7717/peerj.1784)

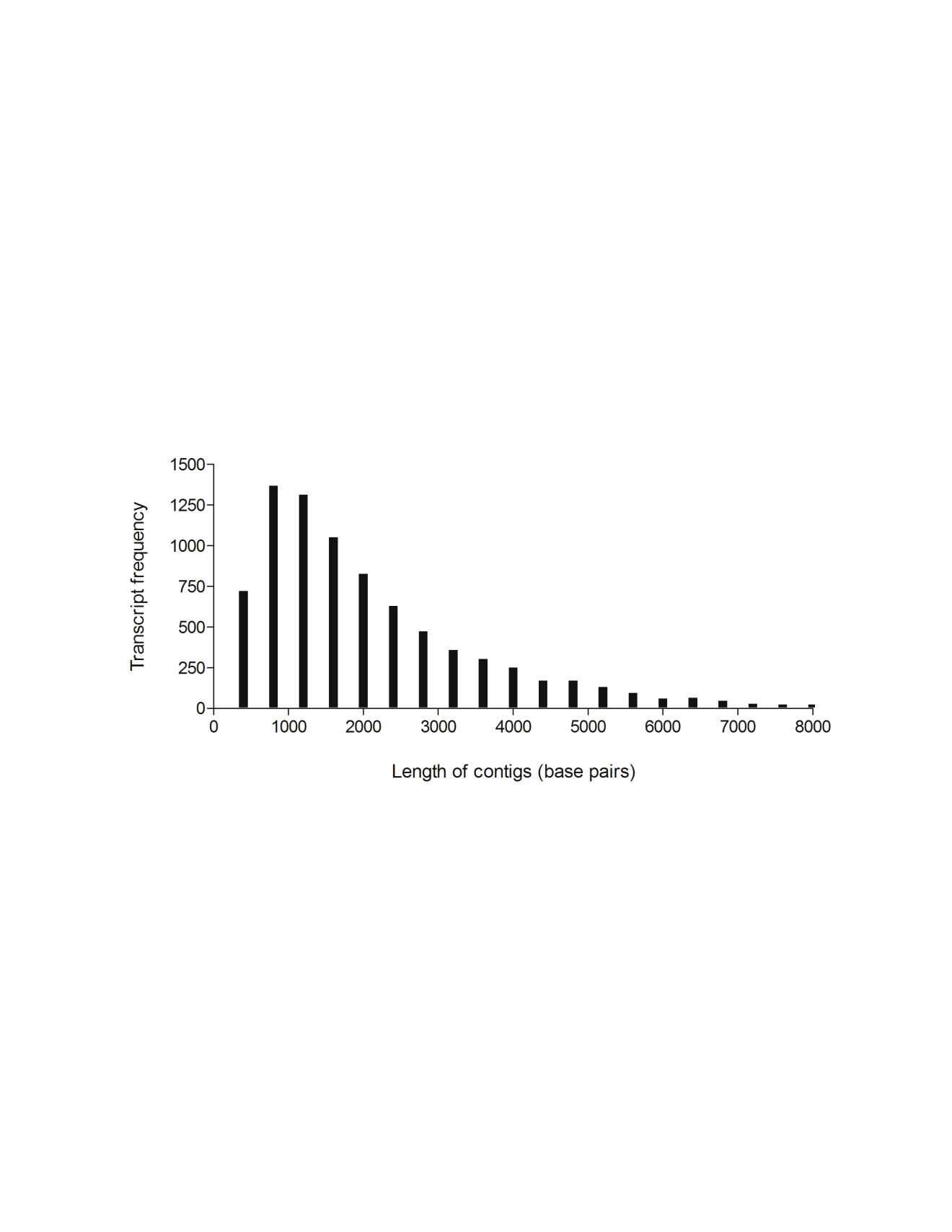


Figure S2. Distribution of contigs by length. Contigs longer than 8000 bp (n = 95) are not shown.

Supplement: Figure S2 — Contigs longer than 8,000 bp (n = 95) are not shown. [file peerj-04-1784-s016.doc]

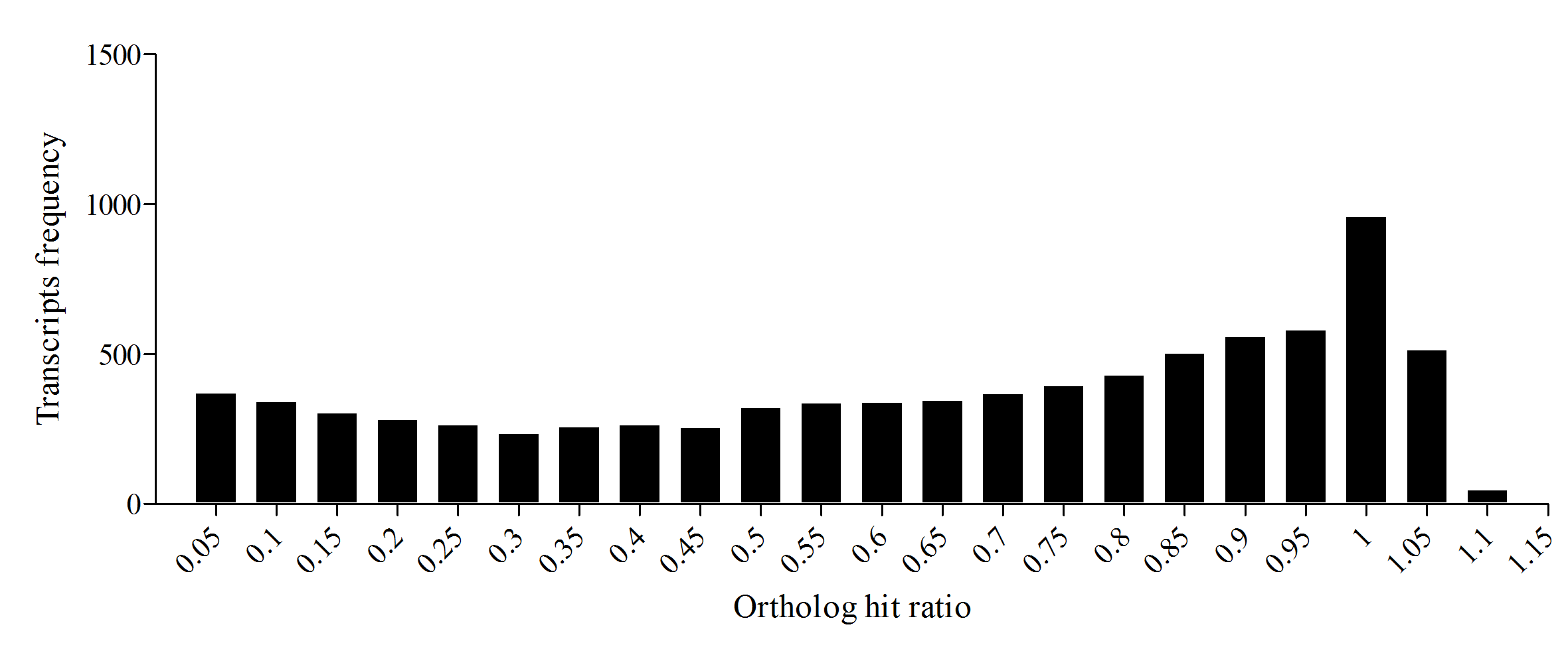


Figure S3. Distribution of ortholog hit ratios for the annotated *Ae. albopictus* transcripts.

Supplement: Figure S3 [file peerj-04-1784-s017.doc]
