# Supplementary material for: A de novo transcriptome of the Malpighian tubules in non-blood-fed and blood-fed Asian tiger mosquitoes Aedes albopictus: insights into diuresis, detoxification, and blood meal processing
Source: PeerJ. 2016 Mar 10;4:e1784. doi: 10.7717/peerj.1784 (PMC4793337; doi:10.7717/peerj.1784)
Supplement: Figure S4 — In the 3 h BF tubules, over 50% of the differentially-expressed transcripts were unique to that time period, whereas in the 12 h and 24 h BF tubules, ∼40% of the differentially-expressed transcripts were unique to their respective period. ‘U’ and ‘D’ indicate numbers of up-regulated and down-regulated transcripts, respectively. BF, blood fed. The Venn diagram was generated using Venny 2.0 (Oliveros, 2007–2015). [file peerj-04-1784-s018.doc]

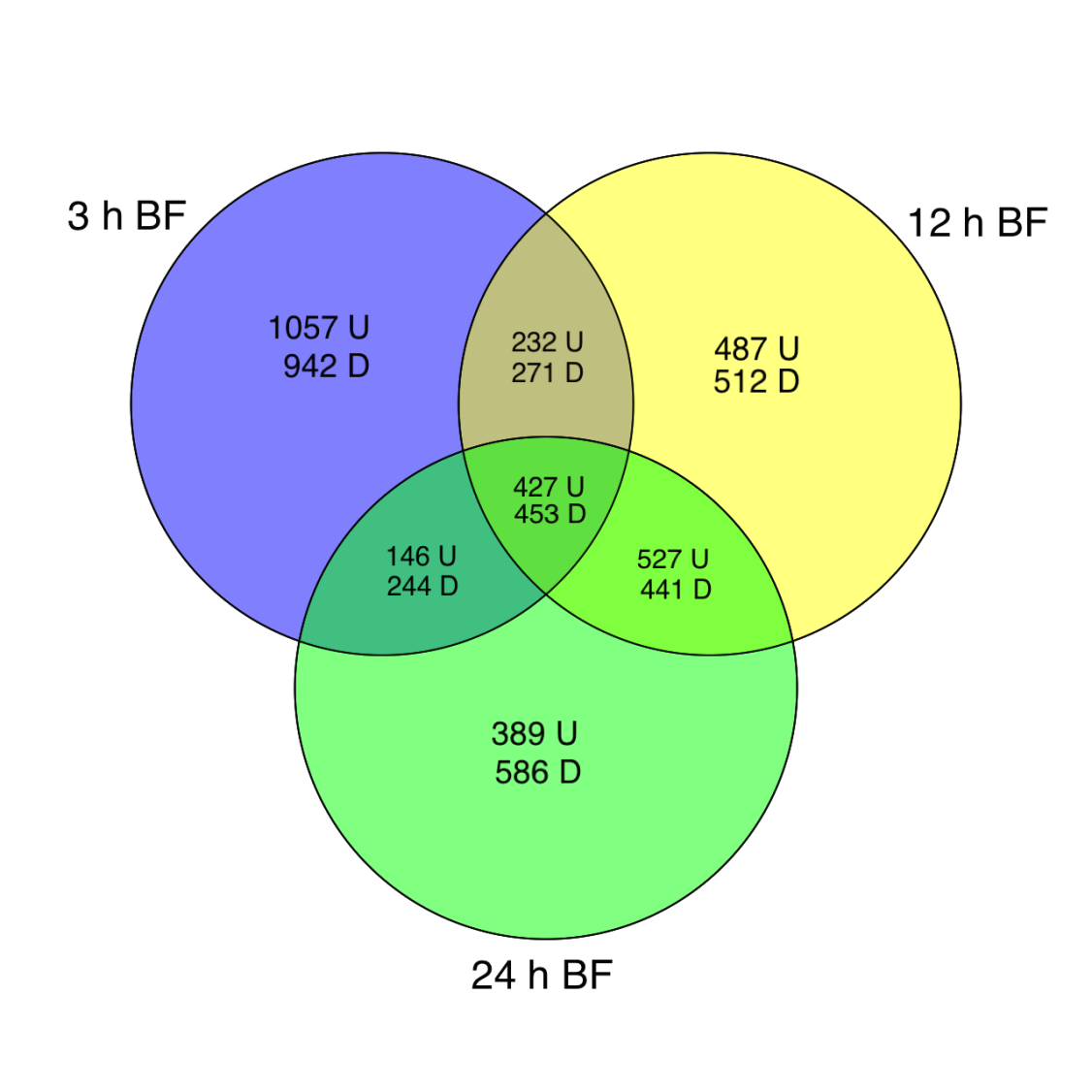


Figure S4. Venn diagram showing the relationships of differentially-expressed transcripts in the Malpighian tubules of BF mosquitoes. In the 3 h BF tubules, over 50% of the differentially-expressed transcripts were unique to that time period, whereas in the 12 h and 24 h BF tubules, ~40% of the differentially-expressed transcripts were unique to their respective period. ‘U’ and ‘D’ indicate numbers of up-regulated and down-regulated transcripts, respectively. BF = blood fed. The Venn diagram was generated using Venny 2.0 (Oliveros, 2007-2015).
