# Supplementary material for: A de novo transcriptome of the Malpighian tubules in non-blood-fed and blood-fed Asian tiger mosquitoes Aedes albopictus: insights into diuresis, detoxification, and blood meal processing
Source: PeerJ. 2016 Mar 10;4:e1784. doi: 10.7717/peerj.1784 (PMC4793337; doi:10.7717/peerj.1784)
Supplement: Figure S5 — See text for details. Gap junctions (innexins) form connections between principal cells and between principal and stellate cells, where they may synchronize the epithelial cells and contribute to the metabolic regulation of fluid secretion (Beyenbach & Piermarini, 2011; Piermarini & Calkins, 2014). The receptors for DH31 and kinin are G protein-coupled receptors (GPCRs) that localize to the basolateral membranes of principal and stellate cells, respectively (Halberg et al., 2015; Kersch & Pietrantonio, 2011; Kwon et al., 2012; Lu, Kersch & Pietrantonio, 2011); transcripts encoding DH44 GPCRs are expressed in the Malpighian tubules of Ae. aegypti and An. gambiae, but have yet to be localized (Jagge & Pietrantonio, 2008; Overend et al., 2015). AE, anion exchanger; AQP, aquaporin; ATP, adenosine triphosphate; DH, diuretic hormone; KCC, K,Cl cotransporter; Kir, inward rectifier K+ channel; NDAE, Na-driven anion exchanger; NHA, Na/H antiporter; NHE, Na/H exchanger; NKA, Na, K-ATPase; NKCC, Na,K,Cl cotransporter; SJ, septate junction. [file peerj-04-1784-s019.doc]

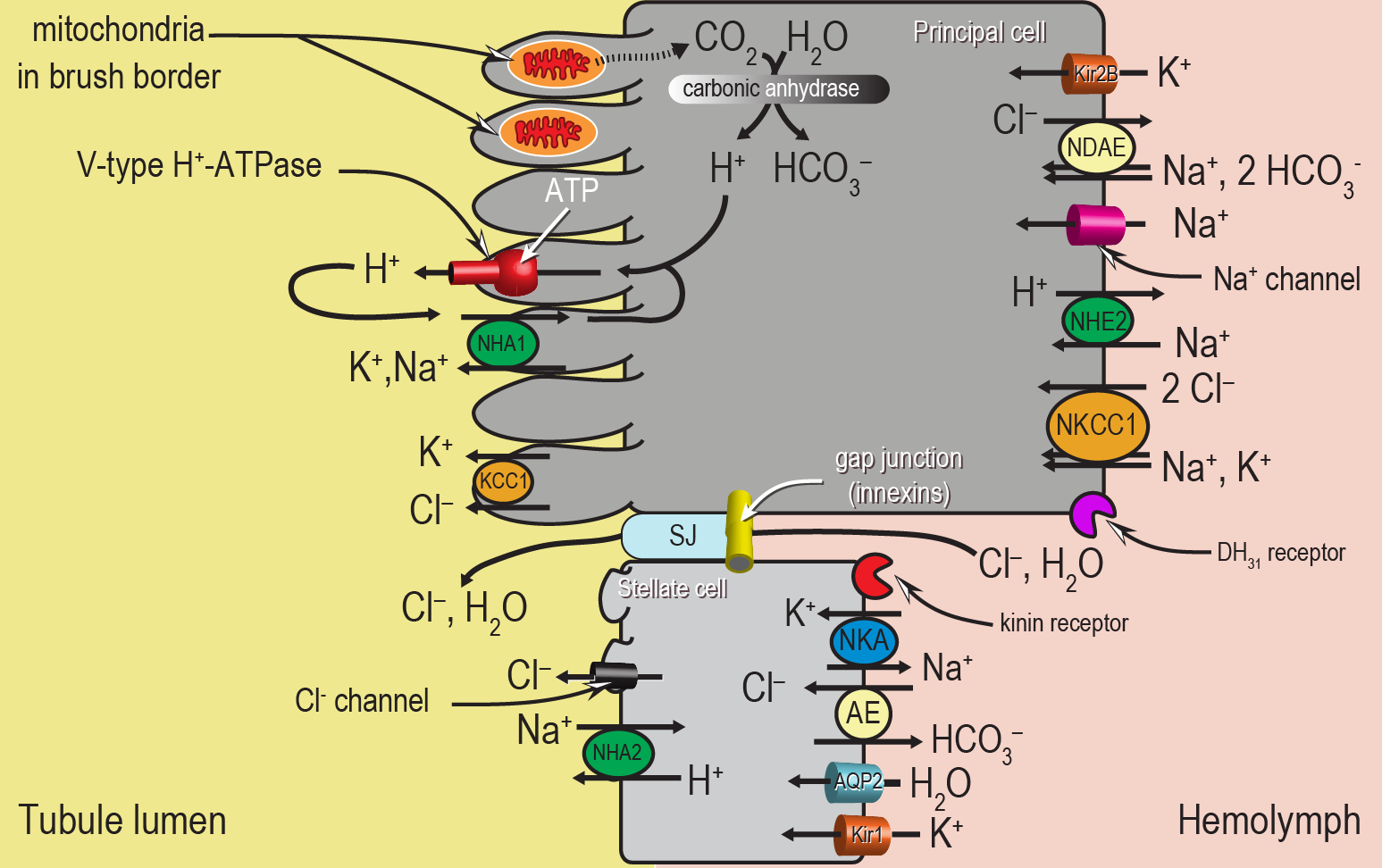


Figure S5. Molecular model of transepithelial fluid secretion in mosquito Malpighian tubules. See text for details. Gap junctions (innexins) form connections between principal cells and between principal and stellate cells, where they may synchronize the epithelial cells and contribute to the metabolic regulation of fluid secretion (Beyenbach & Piermarini, 2011; Piermarini & Calkins, 2014). The receptors for DH_31_ and kinin are G protein-coupled receptors (GPCRs) that localize to the basolateral membranes of principal and stellate cells, respectively (Halberg et al., 2015; Kersch & Pietrantonio, 2011; Kwon et al., 2012; Lu et al., 2011); transcripts encoding DH_44_ GPCRs are expressed in the Malpighian tubules of *Ae. aegypti* and *An. gambiae*, but have yet to be localized (Jagge & Pietrantonio, 2008; Overend et al., 2015). AE, anion exchanger; AQP, aquaporin; ATP, adenosine triphosphate; DH, diuretic hormone; KCC, K,Cl cotransporter; Kir, inward rectifier K^+^ channel; NDAE, Na-driven anion exchanger; NHA, Na/H antiporter; NHE, Na/H exchanger; NKA, Na,K-ATPase; NKCC, Na,K,Cl cotransporter; SJ, septate junction.
