# Supplementary material for: A de novo transcriptome of the Malpighian tubules in non-blood-fed and blood-fed Asian tiger mosquitoes Aedes albopictus: insights into diuresis, detoxification, and blood meal processing
Source: PeerJ. 2016 Mar 10;4:e1784. doi: 10.7717/peerj.1784 (PMC4793337; doi:10.7717/peerj.1784)
Supplement: Figure S6 — Adenosine triphosphate (ATP) that is generated by the mitochondrion is shuttled to the V-type H+-ATPase and provides energy for the translocation of H+ across the apical membrane. The resulting adenosine diphosphate (ADP) is recycled by the mitochondrion. Redrawn and modified from Beyenbach (2001). NHA,Na/H antiporter. [file peerj-04-1784-s020.doc]

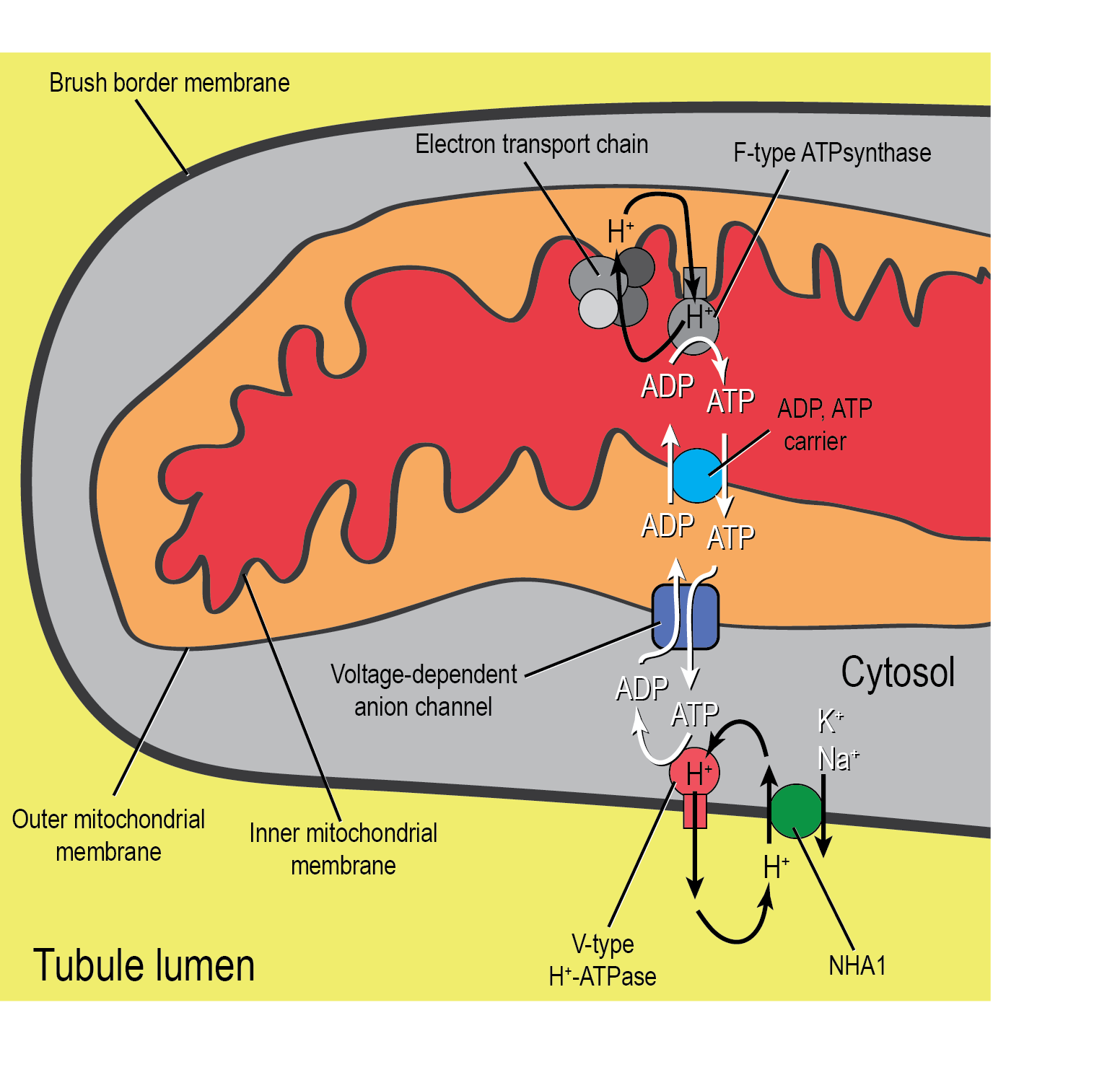


Figure S6. Schematic of functional/metabolic coupling between a mitochondrion and the V-type H^+^-ATPase in an apical microvillus of the principal cell brush border. Adenosine triphosphate (ATP) that is generated by the mitochondrion is shuttled to the V-type H^+^-ATPase and provides energy for the translocation of H^+^ across the apical membrane. The resulting adenosine diphosphate (ADP) is recycled by the mitochondrion. Redrawn and modified from Beyenbach (2001). NHA, Na/H antiporter.
