# Supplementary material for: A de novo transcriptome of the Malpighian tubules in non-blood-fed and blood-fed Asian tiger mosquitoes Aedes albopictus: insights into diuresis, detoxification, and blood meal processing
Source: PeerJ. 2016 Mar 10;4:e1784. doi: 10.7717/peerj.1784 (PMC4793337; doi:10.7717/peerj.1784)
Supplement: Figure S7 — Numbers indicate KEGG enzyme codes corresponding to transcripts that were expressed in Malpighian tubules of NBF mosquitoes (see Table S12 for transcripts that correspond to enzyme codes). Dashed lines indicate potential enzymatic reactions for which no corresponding transcripts were detected in the transcriptome. AMP, Adenosine monophosphate; ADP, adenosine diphosphate; dAMP; deoxyadenosine monophosphate; GMP, guanosine monophosphate; GDP, guanosine diphosphate; dGMP, deoxyguanosine monophosphate; IMP, inosine monophosphate; XMP, xanthosine monophosphate. Redrawn and modified from Ramsey et al. (2010). [file peerj-04-1784-s021.doc]

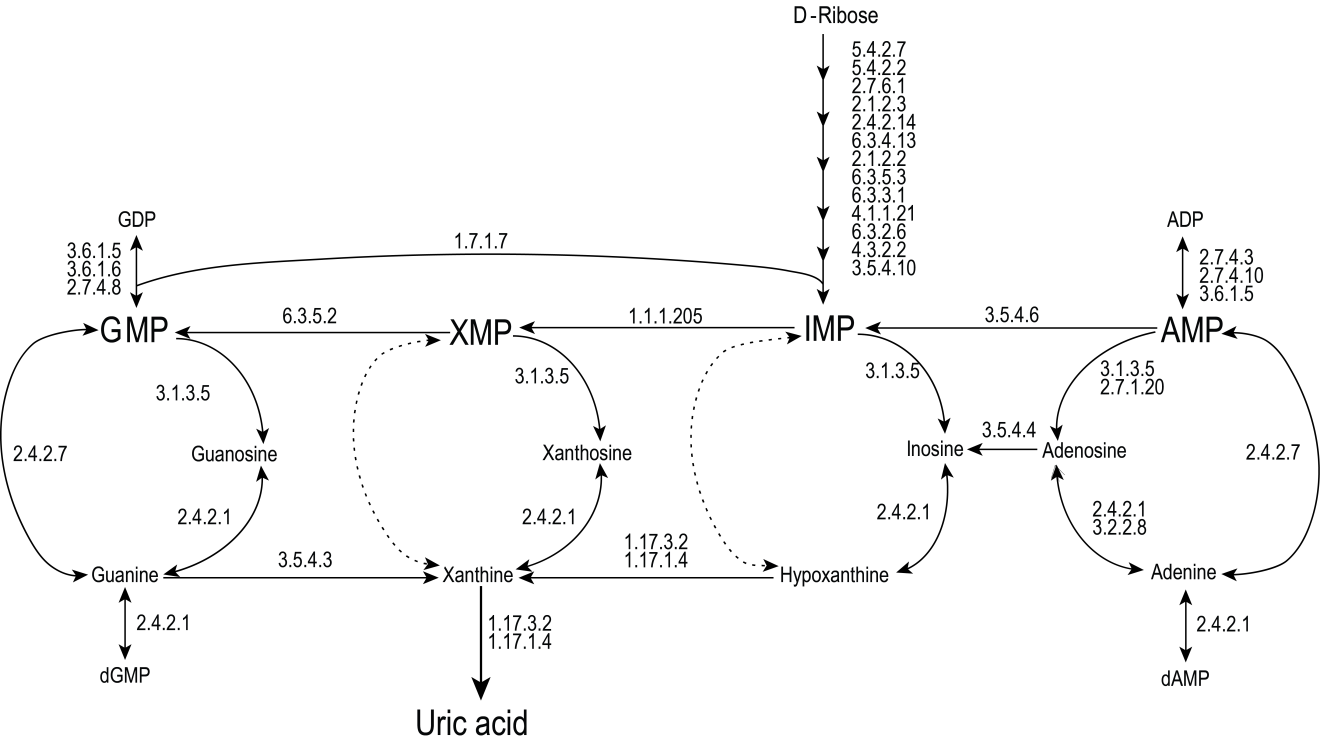


Figure S7. Simplified KEGG pathway of purine metabolism, resulting in uric acid production. Redrawn and modified from (Ramsey et al., 2010). Numbers indicate KEGG enzyme codes corresponding to transcripts that were expressed in Malpighian tubules of NBF mosquitoes (see Table S12 for transcripts that correspond to enzyme codes). Dashed lines indicate potential enzymatic reactions for which no corresponding transcripts were detected in the transcriptome. AMP, Adenosine monophosphate; ADP, adenosine diphosphate; dAMP; deoxyadenosine monophosphate; GMP, guanosine monophosphate; GDP, guanosine diphosphate; dGMP, deoxyguanosine monophosphate; IMP, inosine monophosphate; XMP, xanthosine monophosphate. Redrawn and modified from (Ramsey et al., 2010).
